# Sakuranetin, a Natural Flavonoid, Promising to Manage Grapevine Diseases

**DOI:** 10.3390/molecules31081368

**Published:** 2026-04-21

**Authors:** Corentin Griffon, Brice Dautruche, Bilal Loumi, Abdouramane Dosso, David Lesur, Emilie Isidore, Morad Chadni, Florent Allais, Christian Magro, Charles Monteux, Florence Fontaine

**Affiliations:** 1 Induced Resistance and Plant Bioprotection Research Unit, University of Reims Champagne-Ardenne, UMR INRAE 1488, 51687 Reims, France; corentin.griffon2@etudiant.univ-reims.fr (C.G.); brice.dautruche@chestnut-co.com (B.D.); 2 Chestnut, 26 Rue Barthélémy de Laffemas, 26000 Valence, France; christian.magro@chestnut-co.com (C.M.); charles.monteux@chestnut-co.com (C.M.); 3 URD Agro-Biotechnologies Industrielles (ABI), CEBB, AgroParisTech, 51110 Pomacle, France; bilal.loumi@agroparistech.fr (B.L.); abdouramane.dosso@agroparistech.fr (A.D.); emilie.isidore@agroparistech.fr (E.I.); morad.chadni@agroparistech.fr (M.C.); florent.allais@agroparistech.fr (F.A.); 4 Laboratoire de Glycochimie et des Agroressources, University of Picardie Jules Verne, 33 Rue Saint Leu—UFR des Sciences, 80000 Amiens, France; david.lesur@u-picardie.fr

**Keywords:** plant secondary metabolites, flavanones, grey mold, downy mildew, sustainable crop protection, biocontrol

## Abstract

*Botrytis cinerea* and *Plasmopara viticola*, the causal agents of grey mold and downy mildew, respectively, are two major grapevine pathogens whose control largely relies on synthetic fungicides, raising environmental and health concerns. Plant-derived secondary metabolites, particularly flavonoids involved in plant defense, represent promising sustainable alternatives. Among them, sakuranetin, a flavanone aglycone known for its antifungal activity in rice, remains poorly explored for grapevine protection. In this study, sakuranetin was purified from cherry branches (48 mg) and structurally characterized using UHPLC-ESI-QTOF-MS and NMR analyses. Its antifungal activity against *B. cinerea* and *P. viticola* was evaluated through in vitro, in vivo and *in planta* assays. For *B. cinerea,* our results showed a significant in vitro inhibition of mycelium growth, with EC_50_ values of 16.43 mg·L^−1^, while no protection of detached berries was observed. Against *P. viticola*, sakuranetin has no effect on the release of zoospores, but there is a total inhibition of spore germination at 1 mg·L^−1^ in vitro, confirmed in vivo on a foliar disc. *In planta*, no significant protection is observed at 25 mg·L^−1^, even if some targeted defense genes are induced. Further studies are needed to determine the best concentration of sakuranetin to use to manage *B. cinerea* and *P. viticola in planta*.

## 1. Introduction

Grapevines (*Vitis vinifera* L.) are a crop of major economic importance, cultivated for the production of table grapes, raisins and, mainly, wine grapes [1]. Each year, global grape production reaches approximately 78 million tons over 7.1 million hectares [2]. Grapevines host a wide range of pathogens, among which fungal and oomycete species are the most damaging, causing major economic losses [3,4]. In particular, *Plasmopara viticola*, responsible for downy mildew, and *Botrytis cinerea*, the causal agent of grey mold [5], can cause yield losses of up to 75% [6] and 50% [7], respectively. The control of these diseases still relies heavily on synthetic chemicals [8], despite their well-documented adverse effects on human health and the environment [9,10,11]. In addition, the repeated use of fungicides with a single mode of action promotes the emergence of resistant pathogen strains [12]. Copper-based products remain among the main control strategies for grey mold and downy mildew [13,14], particularly in organic viticulture. However, copper is no longer considered a sustainable solution due to its accumulation in soils, toxicity to non-target organisms, and persistence, which leads to residues in grapes and wine [15]. Regulatory restrictions have further reduced the number of available active substances, increasing risks regarding sustainable disease management [16,17,18]. In this context, the development of environmentally friendly alternatives is urgently needed. Biocontrol strategies, based on natural mechanisms such as competition, antimicrobial metabolite production or induction of plant defense, represent promising solutions [19,20]. Among these approaches, the use of plant-derived natural compounds is particularly attractive (19). Medicinal and aromatic plants are rich sources of secondary metabolites, including flavonoids, which play key roles in plant defense and stress adaptation [21,22,23,24,25]. Flavonoids are one of the most widespread classes of plant secondary metabolites, with more than 9000 structures described to date [26,27]. Structural modifications such as *O*-methylation enhance their physicochemical properties, including stability and lipophilicity, thereby increasing their antimicrobial activity [28,29,30]. Sakuranetin (4′,5-dihydroxy-7-methoxyflavanone), a methylated flavonoid first described in 1908 in the cortex of the cherry tree bark (*Prunus* spp.) [31], is a well-known phytoalexin involved in resistance to several fungal and bacterial pathogens [32,33,34,35]. It also plays an important role in the biotic stress responses of woody species and exhibits strong antifungal activity against wood-decaying fungi [36,37,38]. Despite its promising properties, no studies have yet investigated the effects of sakuranetin against *B. cinerea* and *P. viticola* in grapevine. This study therefore aims to evaluate the protective potential of sakuranetin against these two major grapevine pathogens. Sakuranetin was first purified from *Prunus avium* branch extracts, an important co-product in arboriculture and widely available. Then, the protection in vivo and *in planta* is evaluated against both *P. viticola* and *B. cinerea,* and, finally, its mode of action is investigated.

## 2. Results

### 2.1. Purification of Sakuranetin from Cherry Branch Extract

**Figure 1:**
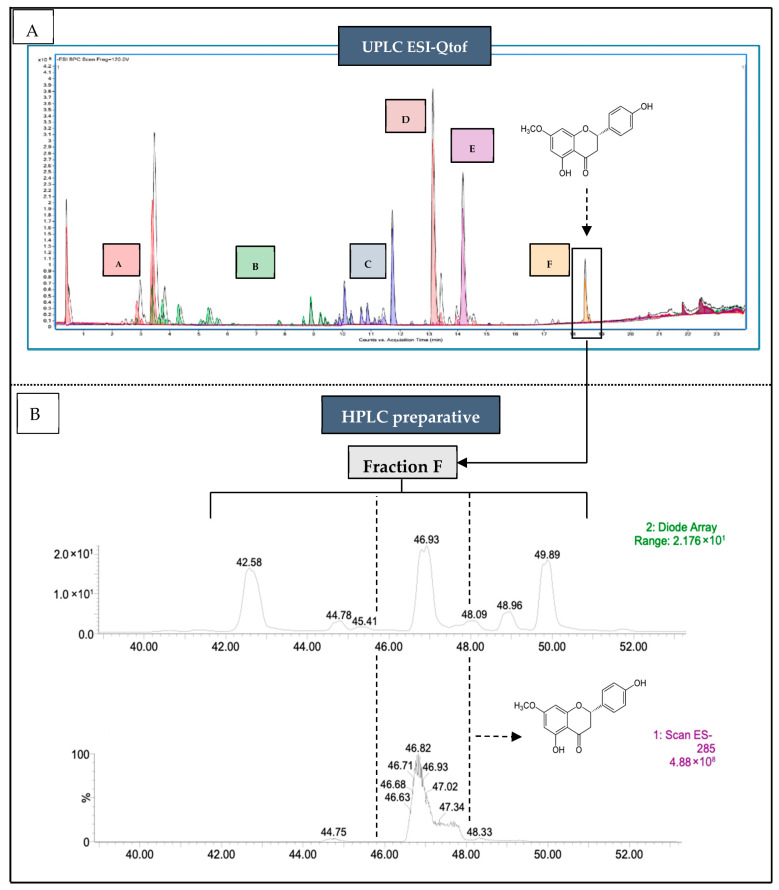
Chromatographic analysis and purification of sakuranetin isolated from the cherry tree branch. (**A**) UPLC-ESI-QTOF-MS chromatogram of the crude extract. The colored areas correspond to the fractions obtained by flash chromatography. Fraction F (highlighted in black) was identified as the target fraction containing sakuranetin, based on its characteristic mass spectrum. (**B**) Preparative HPLC chromatograms of fraction F. The first chromatogram (UV diode-array detection) shows the composition of fraction F with multiple compounds, while the second chromatogram (negative ion mode MS) focuses on the mass of sakuranetin, indicating the elution zone collected to obtain the purified compound.

From the crude extract of cherry branches (*Prunus avium* var. burlat), ~50 mg of sakuranetin was purified. The compound was identified by mass spectrometry, showing a retention time identical to the analytical standard (Figure 1A), with an estimated concentration of 15 ± 2 mg·g^−1^ dry matter. Flash chromatography localized sakuranetin in fraction F, yielding 12 ± 2 mg·g^−1^ of dry extract. This step was repeated five times before preparative HPLC purification, resulting in 48 mg of sakuranetin (Figure 1B). In negative ESI-MS mode, the [M–H]^−^ ion at *m*/*z* 285.1 matched the theoretical mass (C_16_H_14_O_5_; 286.28 g·mol^−1^). Retention time and chromatographic profile were identical to the commercial standard. Structural identity was confirmed by NMR spectroscopy. ^1^H NMR (300 MHz, acetone-d_6_) showed characteristic signals at δ 2.75 and 3.22 (CH_2_), 3.85 (OCH_3_), 5.47 (H-2), aromatic protons between 6.05–7.41, and phenolic protons at 8.50 and 12.14. ^13^C NMR (75 MHz, acetone-d_6_) confirmed the flavanone skeleton. Signal assignments were validated by 2D NMR (COSY, HSQC, HMBC) for both the standard and purified compound, while the DEPT-135 experiment was performed only on the purified sakuranetin. Complete overlap with the standard spectra confirmed purity and identity (Supporting Information, Figures S1–S6).

### 2.2. In Vitro Inhibition of Sakuranetin Against P. viticola and B. cinerea

**Figure 2:**
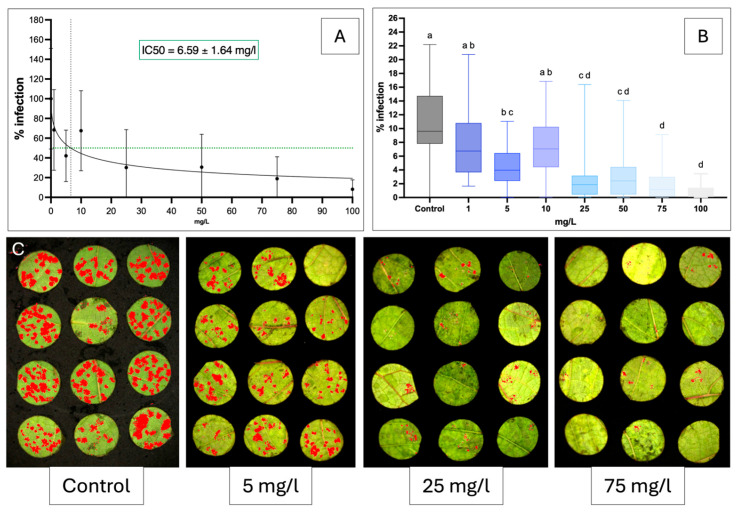
Effect of sakuranetin against *Plasmopara viticola* under in vitro conditions. Data represent the mean ± SD of three independent biological replicates. (**A**) Non-linear regression curve used to estimate the IC50, where the intersection point of the dashed lines corresponds to the IC50 value. (**B**) Representation of the same data as box plots, where the letters above the boxes indicate statistically significant differences between treatments. (**C**) Representative illustration of leaf discs from Vitis vinifera cv. Chardonnay treated with different concentrations of sakuranetin (0, 5, 25 and 75 mg·L^−1^) and then inoculated with *P. viticola*. Sporulation areas are highlighted in red after image analysis.

**Figure 3:**
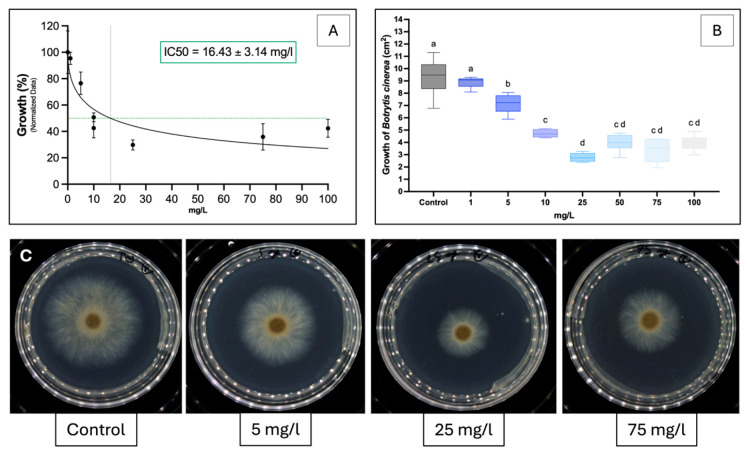
Antifungal effect of sakuranetin against *Botrytis cinerea* under in vitro conditions. Data represent the mean ± SD of three independent biological replicates. (**A**) Non-linear regression curve used to estimate the IC50, where the intersection point of the dashed lines corresponds to the IC50 value. (**B**) Representation of the same data as box plots, where the letters above the boxes indicate statistically significant differences between treatments. (**C**) Representative illustration of *B. cinerea* growth at 3 days in Petri dishes treated with different concentrations of sakuranetin (0, 5, 25 and 75 mg·L^−1^).

For *P. viticola*, non-linear regression showed a clear dose–response relationship, with an IC_50_ of 6.59 mg·L^−1^ (Figure 2A). Significant inhibition occurred from 5 mg·L^−1^ (*p* < 0.05), with concentrations of up to 100 mg·L^−1^ progressively reducing sporulation (Figure 2B), reaching ~90% inhibition at 25 mg·L^−1^. Visual observations confirmed strong inhibition at 25 mg·L^−1^ and little to no sporulation at 75 mg·L^−1^ (Figure 2C). For *B. cinerea*, the IC_50_ was 16.43 mg·L^−1^ (Figure 3A). Mycelial growth was significantly inhibited from 5 mg·L^−1^, with a dose-dependent decrease up to 100 mg·L^−1^ (Figure 3A,B). A plateau was reached above 25 mg·L^−1^, indicating no further increase in activity, as confirmed visually (Figure 3C). At all concentrations, sakuranetin exhibited a fungistatic rather than a fungicidal effect.

### 2.3. No Antifungal and Anti-Oomycete Activity of Sakuranetin in Planta and In Vivo

**Figure 4:**
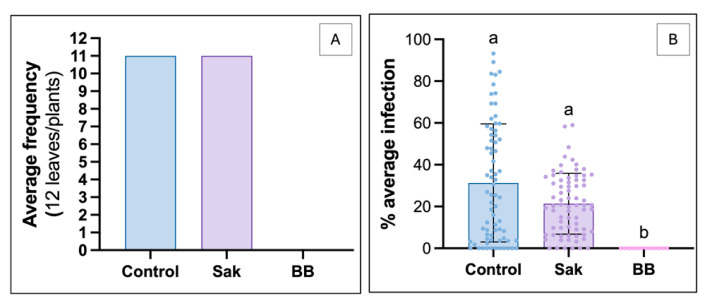
Results of the *in planta* trial on in vitro plants acclimatised under different conditions. Sakuranetin 25 mgL (Sak), positive control (BB = Bordeaux mixture). (**A**) The frequency of infection by *Plasmopara viticola* (number of leaves affected/total number of leaves) and (**B**) the intensity of infection, quantified by image analysis using the TWS plugin. The letters indicate statistically distinct groups. The dots superimposed on the bars correspond to the individual values obtained for each leaf, allowing the distribution and variability of the data within each treatment to be visualised.

**Figure 5:**
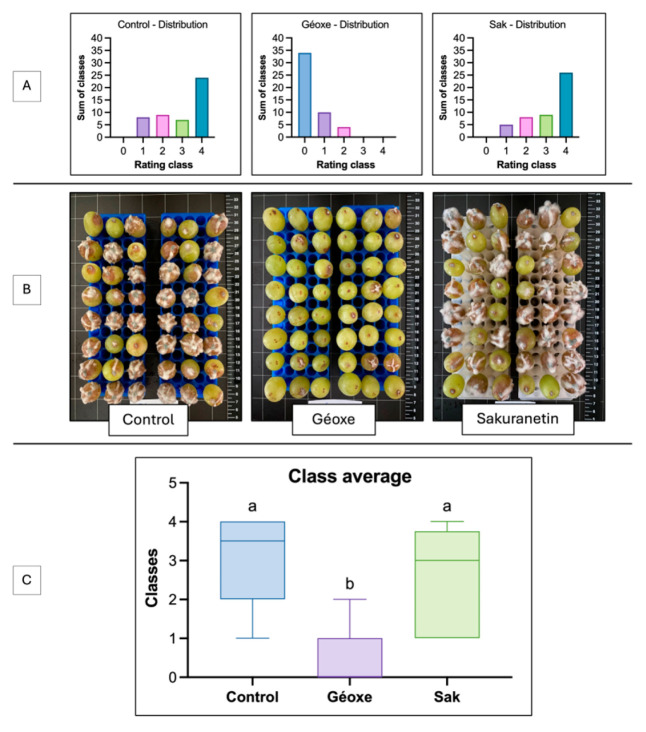
In vivo evaluation of the efficacy of sakuranetin against *Botrytis cinerea* on detached berries. (**A**) Distribution of fruit according to their rating class (0 to 4) for each treatment: negative control, positive control (Geoxe WG) and sakuranetin (25 mg·L^−1^). (**B**) Photographic illustrations of the condition of the fruit after the incubation period. (**C**) Box plots showing the mean classes for each treatment. Different letters (a, b) indicate significant differences.

The concentration of 25 mg·L^−1^ sakuranetin was selected based on the in vitro plateau (~80% inhibition), but this efficacy was not reproduced *in planta* against *P. viticola*. Both control and treated plants showed high infection levels, with up to 11 infected leaves out of 12 (Figure 4A), and similar infection intensity, unlike the positive control (Figure 4B). However, infection values under sakuranetin were more homogeneous and slightly shifted toward lower intensities, as confirmed by the Kolmogorov–Smirnov test (*p* = 0.0013). For *B. cinerea*, in vivo assays on detached berries also failed to confirm in vitro activity. Both control and treated berries showed high proportions of severe infection (class 4), whereas those treated with Géoxe were mostly uninfected (class 0) (Figure 5A,C). Visual assessments confirmed the absence of a protective effect at 25 mg·L^−1^ (Figure 5B).

### 2.4. Anti-Germinative Action and Mycelial Growth Inhibitor of Sakuranetin

**Figure 6:**
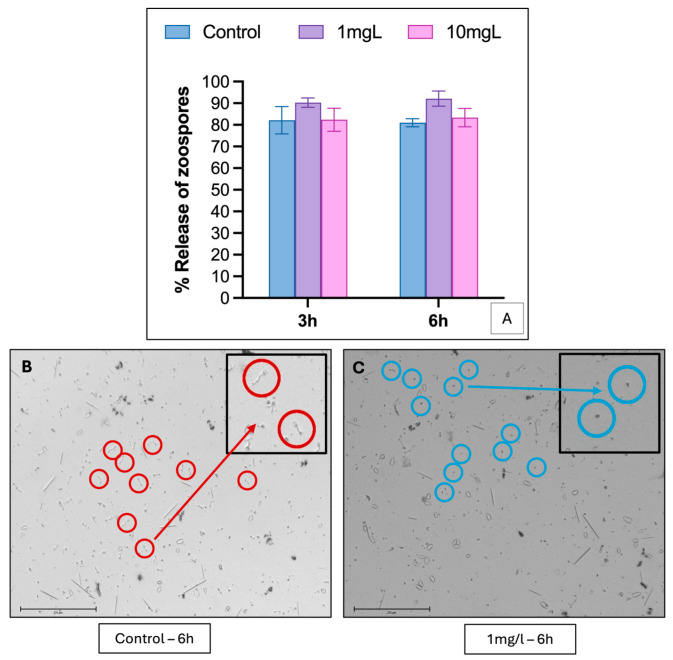
Effect of sakuranetin on the release and germination of *Plasmopara viticola* zoospores. (**A**) Average percentage of zoospore release measured after 3 h and 6 h of incubation, expressed relative to the control (100%). (**B**,**C**) Microscopic observations of zoospore germination after 6 h of incubation, in the absence (**B**, control) or presence of sakuranetin at 1 mg·L^−1^ (**C**). The circles indicate the spores observed, and the boxes (top right) show a magnification illustrating the presence or absence of germination tubes. (**B**) Control: spores with a well-developed germination tube. (**C**) Sakuranetin (1 mg·L^−1^): spores without germination tubes, indicating complete inhibition of germination. Scale: 275 µm.

**Figure 7:**
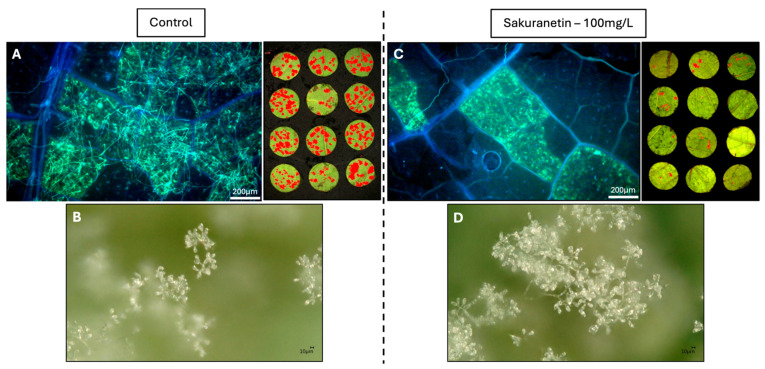
Observation of *Plasmopara viticola* development 7 days after inoculation. (**A**,**C**) Epifluorescence microscopy (×4): visualization of the mycelial network stained with aniline blue (**left**) and corresponding macroscopic appearance of leaf discs prior to discoloration (**right**), where downy mildew infection is highlighted in red. (**B**,**D**) 3D microscopy (×100): visualization of sporangiophores bearing sporangia.

No significant effect of sakuranetin on *P. viticola* zoospore release was observed at 3 or 6 h (Figure 6A). However, zoospore germination was completely inhibited at 1 mg·L^−1^, without affecting motility (Figure 6B,C). *In planta*, epifluorescence microscopy showed extensive mycelial development and sporulation in controls (Figure 7A), whereas sakuranetin (25 mg·L^−1^) moderately reduced mycelial growth, with lower fluorescence intensity and smaller sporulation areas (Figure 7C). The presence of sporangiophores and sporangia remained similar in treated and control tissues, indicating no effect on their formation (Figure 7B,D).

### 2.5. Sakuranetin Induces the Expression of Some Defence Genes of Vitis vinifera

**Figure 8:**
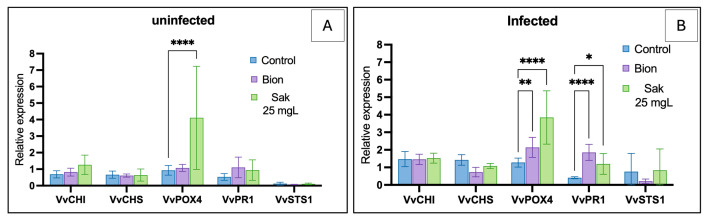
Relative expression of defense-related genes in acclimated *Vitis vinifera* (cv. Chardonnay) plantlets under *in planta* conditions: (**A**) uninfected and (**B**) infected with *Plasmopara viticola*. Transcript levels of *VvCHI*, *VvCHS*, *VvPOX4*, *VvPR1*, and *VvSTS1* were quantified by RT-qPCR under three treatments: negative control (aqueous solution with 1% ethanol), positive control (Bion^®^ 2 g·L^−1^), and sakuranetin (25 mg·L^−1^). Data represent mean relative expression ± standard error (SE), normalized to the negative control. Statistical differences are indicated relative to the negative control. Asterisks denote significance levels: *p* < 0.05 (*); *p* < 0.01 (**); *p* < 0.0001 (****); If no symbol is shown, the result is considered not significant.

In uninfected plantlets, sakuranetin (25 mg·L^−1^) significantly induced *VvPOX4* expression compared with both the water control and Bion^®^ (4.11-fold, *p* ≤ 0.05; Figure 8A), while *VvCHI*, *VvCHS*, *VvWRKY1* and *VvSTS1* were unchanged. In infected plantlets, *VvPOX4* was also significantly upregulated (3.85-fold, *p* ≤ 0.05), reaching levels comparable to Bion^®^ (Figure 8B). *VvPR1* showed a slight increase under sakuranetin (1.20-fold, *p* ≤ 0.05) and Bion^®^, whereas *VvCHI*, *VvCHS* and *VvSTS1* remained unaffected. Overall, *VvPOX4* displayed the strongest and most consistent induction, while other genes showed limited or no response.

## 3. Discussion

Sakuranetin is widely recognized as a key component of plant defense against diverse pathogens. In rice, it accumulates in infected tissues and reaches higher levels in resistant cultivars than in susceptible cultivars [39,40,41]. It exhibits strong antifungal activity, inhibiting *Magnaporthe oryzae* growth, spore germination, and germ tube elongation at 100 µM [39], and interferes with clathrin-mediated endocytosis, disrupting effector delivery and enhancing resistance [42]. Its activity extends to other pathogens, improving resistance to *Ustilaginoidea virens* [43] and affecting bacterial species such as *Burkholderia glumae* and *Xanthomonas oryzae* [34]. In other species, UV-induced accumulation enhances resistance to *B. cinerea* [44], and it has been identified in potato cultivars resistant to *Phytophthora infestans* [45]. Transcriptomic analyses further revealed extensive gene reprogramming in *M. oryzae*, suggesting multiple modes of action [46]. Together, these findings highlight sakuranetin as a multifunctional defense molecule combining antimicrobial activity, modulation of defense signaling, and interference with pathogen infection, supporting its evaluation against *P. viticola* and *B. cinerea*.

### 3.1. Variability and Technical Constraints in Sakuranetin Extraction

Sakuranetin and its glycosylated form, sakuranin, are widespread flavonoids found in various plant extracts and derived products such as honey [31]. They are particularly abundant in the genus *Prunus*, especially in cherry species [47]. In this study, sakuranetin was purified from *Prunus avium* branches at 15 ± 2 mg·g^−1^ dry weight, markedly higher than previously reported values (~0.7 mg·g^−1^), from stems [48]. This difference may reflect the predominance of sakuranin and methodological factors [49], but it is more likely due to natural variability in polyphenol content linked to environmental and cultivation conditions [50,51]. An alternative approach could exploit the abundance of sakuranin, which can be converted into sakuranetin by mild acid or enzymatic hydrolysis using β-glycosidases, potentially improving yield.

### 3.2. High Direct Activity of Sakuranetin Against Plasmopara viticola and Botrytis cinerea

This study provides the first evidence of sakuranetin activity against *P. viticola*. In vitro, it showed a low IC_50_ of 6.59 mg·L^−1^ (21 µM; Figure 2), placing it among the most active natural compounds against grapevine downy mildew. Its activity is comparable to stilbene phytoalexins such as ε- and δ-viniferin (IC_50_ = 12 and 18 µM) [52,53,54], whereas flavones from *Glechoma hederacea* showed no activity at 500 mg·L^−1^ [55]. Mechanistically, sakuranetin did not affect zoospore release but completely inhibited germination at 1 mg·L^−1^ (≈3.49 µM), as confirmed by epifluorescence microscopy. Similar effects have been reported for dehydroeffusol, larixyl acetate [56] and larixol [57], indicating comparable or higher activity. Against *B. cinerea*, sakuranetin also showed antifungal activity (IC_50_ = 16.43 mg·L^−1^; 57 µM), without complete inhibition of mycelial growth, suggesting a fungistatic effect. Its activity exceeds that of several terpenes [58] and phenylpropenes [59], but remains slightly lower than δ-viniferin [60]. Compared with most flavonoids, including gnaphaliin A [61], sakuranetin ranks among the most potent. Overall, these results identify sakuranetin as a promising natural antifungal compound active against both *P. viticola* and *B. cinerea*.

### 3.3. Sakuranetin, a Promising Molecule for Protecting Plants, Needs to Be Improved

Although *in vitro* results were promising, they were not confirmed in vivo or *in planta*, as sakuranetin showed no significant protective effect. Against *P. viticola*, a slight inhibitory trend with more homogeneous infection levels was observed (Figure 4), as indicated by the Kolmogorov–Smirnov test, whereas no protection occurred against *B. cinerea* (Figure 5). This discrepancy likely reflects limited bioavailability at the infection site. Antifungal efficacy depends not only on intrinsic activity but also on maintaining sufficient *in planta* concentrations. Several physico-chemical constraints may reduce availability, including limited solubilization, adsorption to cuticular waxes due to lipophilicity [62,63], photodegradation [64], and wash-off under high humidity [65]. Biological and temporal factors may also contribute. Longer infection dynamics *in planta* may reduce efficacy if the compound degrades before key stages such as spore germination. Tissue complexity may further enhance pathogen development compared to simplified *in vitro* systems. In *B. cinerea*, wounding-induced nutrient leakage may have increased infection pressure, masking moderate effects. Consistently, sakuranetin only partially inhibits conidial germination under low nutrient conditions [66]. Detoxification mechanisms may also play a role, as reported for *M. oryzae* and *R. solani* [67,68]. Although not demonstrated in *B. cinerea*, its ability to degrade compounds such as resveratrol [69] and methylated isoflavones [70] suggests that similar processes could occur. Such discrepancies between *in vitro* and *in planta* activity are common and often reflect limits in stability, diffusion, or bioavailability, as reported for *Warburgia ugandensis* [71] and *Larix decidua* [57]. Thus, the lack of *in planta* efficacy does not question sakuranetin intrinsic activity but highlights constraints related to its delivery and persistence. These aspects will be addressed in future studies to optimize its efficacy and further investigate its role in grapevine defense.

### 3.4. Activation of the Targeted Defense Gene of Grapevine by Sakuranetin

Sakuranetin is the main flavonoid phytoalexin in rice, induced by stress and involved in resistance, notably through modulation of pathogen effector endocytosis. Although not identified as a phytoalexin in grapevine, it may act as a signaling or priming compound. We therefore tested whether exogenous sakuranetin could activate grapevine defenses. At 25 mg·L^−1^, sakuranetin induced a response similar to Bion^®^, with significant upregulation of *VvPOX4* and *VvPR1*, while *VvCHI*, *VvCHS*, and *VvSTS1* were unchanged. However, the *VvPR1* increase (~1.2-fold) is likely of limited biological relevance. In non-infected plants, sakuranetin specifically induced *VvPOX4*, suggesting activation of an oxidative response involving class III peroxidases, potentially supported by ROS production, as reported for other flavonoids such as naringenin [72]. The lack of *VvPR1* induction indicates weak activation of the salicylic acid pathway. After *P. viticola* infection, *VvPOX4* remained induced, while *VvPR1* showed only a slight increase, preventing firm conclusions about SA pathway activation. Overall, sakuranetin triggers a partial defense response mainly via oxidative pathways, without conferring effective protection under the tested conditions.

## 4. Materials and Methods

To improve readability while maintaining reproducibility, only the essential methodological details are presented in the main text, whereas full experimental procedures and technical specifications are provided in the Supporting Information (pp. 8–15). Readers are strongly encouraged to consult the Supporting Information for detailed Materials and Methods.

### 4.1. Obtaining Sakuranetin from Plant Material

#### 4.1.1. Extraction and Preparation of the Crude Extract from Cherry Tree Branches

Cherry branches (*Prunus avium* var. burlat) were collected in south-eastern France (Drôme department, Chestnut site, Montélimar; 44°33′31″ N, 04°45′03″ E) and processed as described in [73] with minor modifications. Ground material was extracted by reflux maceration in ethanol/water (70/30, *v*/*v*) at 70 °C for 30 min. The extract was filtered, centrifuged, concentrated under reduced pressure and freeze-dried to obtain the crude extract.

#### 4.1.2. Detection and Identification of Sakuranetin in Crude Cherry Branch Extract

LC–MS analyses were performed using an Agilent 1290 UPLC system coupled to a 6545 Q-ToF mass spectrometer with UV-DAD detection (Agilent Technologies, Santa Clara, CA, USA). Separation was achieved on a C18 column at 40 °C using a water/acetonitrile gradient (0.1% formic acid). MS analyses were conducted in negative ESI mode (*m*/*z* 50–1050). Data were processed with MassHunter (version 12.0, Agilent Technologies, Santa Clara, CA, USA) and compared with internal and public databases (NIST, Metlin). Sakuranetin was identified based on its exact mass ([M–H]^−^ = 285.0763 *m*/*z*) and confirmed using a commercial standard by retention time and MS data comparison.

#### 4.1.3. Two-Step Purifications of Sakuranetin from Crude Cherry Branch Extract

##### Step 1—Fractionation by Flash Chromatography and Identification of the Fraction Containing Sakuranetin:

Preliminary fractionation of the crude extract was performed using a Puriflash PF-5.250-UV800 system (Interchim, Montulçon, France) equipped with a C18 reverse-phase column. For each run, 1 g of crude extract was adsorbed onto celite, dried, and loaded onto a dry-load cartridge. Elution was carried out using water and isopropanol (both containing 0.1% acetic acid) under a stepwise gradient at 50 mL·min^−1^. Fractions were collected based on UV detection (254 and 280 nm) and pooled according to their spectral profiles. The fraction containing sakuranetin was identified by UPLC-ESI-QTOF-MS as described in Section 4.1.2.

##### Step 2—Purification of Sakuranetin by Preparative HPLC from the Fraction of Interest:

Sakuranetin was purified by preparative HPLC using a Waters system equipped with PDA and MS detection (Waters Corporation, Milford, MA, USA). Samples were injected in partial loop mode and separated on a C18 column (250 × 21.2 mm, 5 µm) at 20 mL·min^−1^. The mobile phase consisted of water and methanol (both with 0.1% formic acid) using a linear gradient from 100% A to 100% B. UV detection was monitored between 190 and 400 nm, and MS analyses were performed in negative ESI mode (*m*/*z* 50–500). Sakuranetin was collected based on UV signals and its characteristic [M–H]^−^ ion at *m*/*z* 285.1.

#### 4.1.4. Structural Verification of Purified Sakuranetin

Structural confirmation of purified sakuranetin was performed by NMR spectroscopy to exclude isomeric compounds. ^1^H, ^13^C and 2D NMR experiments (COSY, HSQC, HMBC, DEPT-135) were acquired on a Bruker 300 MHz spectrometer equipped with a cryoprobe (Bruker, Billerica, MA, USA). Samples were dissolved in acetone-d_6_ and analyzed in 3 mm tubes. Data acquisition was performed using TopSpin software version 3.2 (Bruker, Billerica, MA, USA), and spectra were processed and interpreted using MestReNova version 14.0.0-23239 (Mestrelab Research, Santiago de Compostela, Spain). The resulting spectra were compared with those of a commercial sakuranetin standard analyzed under identical conditions, confirming structural identity.

#### 4.1.5. Solvent and Reference Substance

The sakuranetin analytical standard was purchased from Extrasynthese (Lyon, France) and its identity and purity (≥90%) were confirmed by UV–visible diode array spectroscopy, LC–MS and NMR analyses. Acetonitrile used for UPLC analyses was obtained from VWR (VWR international, Radnor, PA, USA), while formic and acetic acids were supplied by Fisher Scientific (Thermo Fishcer Scientific, Waltham, Massachusetts, USA). Ethanol, methanol and isopropanol used for extraction, fractionation and purification were purchased from VWR. Ultrapure water was produced using a Milli-Q Integral 5 system (Merck Millipore, Burlington, MA, USA).

### 4.2. Evaluation of the Protective Activity of Purified Sakuranetin Against Two Major Grapevine Pathogens

#### 4.2.1. Biological Material

For *P. viticola*, both *in vitro* and *in planta* assays were conducted using *Vitis vinifera* cv. Chardonnay plantlets derived from *in vitro* culture and acclimatized in greenhouse conditions. *In vitro* experiments were performed on leaf discs, while *in planta* assays used plantlets at the 12-leaf stage. Inoculation was carried out by spraying a spore suspension (1 × 10^4^ spores·mL^−1^) onto the abaxial leaf surface, followed by incubation at 24 °C (24 h dark, then 16 h/8 h light/dark). *P. viticola* was maintained on sterilized grapevine leaves on agar. *Botrytis cinerea* (strain BC630) was cultured on Potato Dextrose Agar (PDA) from glycerol stocks and incubated at 20 °C in the dark.

#### 4.2.2. *In Vitro* Evaluation of Purified Sakuranetin

For both pathogens, Sakuranetin was prepared at concentrations of 0, 1, 5, 10, 25, 50, 75, and 100 mg·L^−1^ in water containing 1% ethanol. The solutions were kept in an ultrasonic bath until use to maintain the solubility of sakuranetin.

##### Evaluation of Anti-Oomycete Activity Against *Plasmopara viticola*

Leaf discs (15 mm) from acclimatized *Vitis vinifera* cv. Chardonnay plantlets were placed on agar (12 discs per treatment). Sakuranetin was applied 24 h prior to inoculation, with controls receiving solvent only. Discs were inoculated with a *P. viticola* spore suspension (1 × 10^4^ spores·mL^−1^) and incubated (24 h dark, then 7 days at 22 °C, 16 h/8 h light/dark). Experiments were performed in triplicate. Disease development was quantified by image analysis using Fiji version 20250529-2217 (ImageJ distribution, National Institutes of Health, Bethesda, MD, USA) as described in [74] and expressed as percentage inhibition relative to the control.

##### Evaluation of Antifungal Activity Against *Botrytis cinerea*

PDA medium was prepared and supplemented with sakuranetin (1% ethanol final). Mycelial plugs (3 mm) of *B. cinerea* from one-week-old cultures were placed at the center of Petri dishes (6 replicates per treatment) and incubated at 20 °C in the dark for 3 days. Mycelial growth was quantified by image analysis using Fiji and expressed as percentage inhibition relative to the control. Experiments were performed in triplicate.

#### 4.2.3. Confirmation of the Anti-*Plasmopara viticola* Activity *in Planta* of Purified Sakuranetin on Acclimatised Plantlets

##### Greenhouse Experiment and Inoculation with *Plasmopara viticola*

The experiment was conducted in a greenhouse (20–28 °C, natural light) using acclimatized grapevine plantlets. Treatments were divided into two blocks, a protection block (disease assessment) and an elicitor-effect block (leaf sampling), to avoid interference between sampling and disease development. Each treatment included six plants. Preventive applications were performed 24 h before inoculation. *P. viticola* inoculation was carried out by spraying a sporangial suspension (2 × 10^4^ sporangia·mL^−1^), followed by incubation (24 h dark, then 10 days under day/night conditions with high humidity). In the protection block, three treatments were tested: a negative control (water + 1% ethanol), a positive control (Bordeaux mixture), and sakuranetin (25 mg·L^−1^). This concentration corresponded to the onset of the dose–response plateau (~80% efficacy *in vitro*), supporting its relevance for *in planta* evaluation.

##### Evaluation of the Protection, Image Processing and Data Acquisition

After incubation, leaves were collected, placed in moist Petri dishes, and stored at 4 °C prior to imaging. The abaxial surface was photographed, and disease development was quantified using Fiji version 20250529-2217 (ImageJ distribution, National Institutes of Health, Bethesda, MD, USA) with thresholding based on Trainable Weka Segmentation (TWS). Images were pre-processed (contrast enhancement, gradient filtering with MorphoLibJ) before analysis. The model, trained on representative leaves, included four classes (background, leaf tissue, sporulation, veins) (Supporting Information, Figure S8). Infection was quantified in the protection block as the proportion of leaf area covered by sporulation relative to total leaf surface.

#### 4.2.4. Confirmation of the In Vivo Anti-*Botrytis cinerea* Activity of Purified Sakuranetin on Detached Berries

##### Plant Material/Detached Berries

Grape bunches (white Muscat table variety) were commercially sourced. Berries were detached with pedicels, surface-sterilized in 70% ethanol, rinsed with sterile water, and placed on racks with pedicels inserted into water to maintain physiological conditions. Each treatment included 48 berries.

##### Treatments and Inoculation of *Botrytis cinerea*

Treatments were applied preventively 24 h before inoculation by spraying the berries, followed by drying and a 24 h incubation period. Solutions were prepared in water containing 1% ethanol, and sakuranetin was applied at 25 mg·L^−1^. A commercial fungicide (Géoxe WG^®^, fludioxonil) was used as a positive control. Berries were artificially wounded (two punctures per berry) to facilitate infection [75], and each wound was inoculated with a spore suspension (1 × 10^6^ spores·mL^−1^). Samples were incubated for 7 days under high humidity (16 h/8 h light/dark).

##### Quantification and Evaluation of the Protection

To quantify the protection, a rating scale was established to classify each berry according to its degree of infection, thereby enabling an objective comparison of the different modalities (Supporting information; Figure S7).

### 4.3. Elucidation of Protective Activity Through Mechanistic Study of Purified Sakuranetin

#### 4.3.1. Microscopic Observation at Different Stages of the *Plasmopara viticola* Infection Cycle

##### Impact on Sporangia Release, Motility and Spore Germination:

A spore suspension was mixed with sakuranetin (1, 10 and 100 mg·L^−1^; final spore concentration 1 × 10^4^ spores·mL^−1^, 1% ethanol) in 96-well plates. Each condition was tested in six replicates. Observations were performed by optic microscopy EVOS M7000 (Thermo Fishcer Scientific, Waltham, Massachusetts, USA) at ×20 at 0, 3 and 6 h, and images were analyzed using Fiji version 20250529-2217 (ImageJ distribution, National Institutes of Health, Bethesda, MD, USA). Zoospore release was expressed as the percentage of empty sporangia, while germination was assessed qualitatively based on germ tube formation.

##### Impact on Sporangiophore Development:

Sporulation of *P. viticola* was observed on leaf discs using a 3D digital microscope Keyence VHX-7000, (Keyence Corporation, Osaka, Japan) at ×100–×400 magnification. Images were acquired with VHX software version 1.3 (Keyence Corporation, Osaka, Japan) and adjusted for brightness and contrast only. Observations were performed on at least six leaf discs per condition and were representative of the results.

##### Impact on Mycelial Network Development:

Leaf discs were observed by epifluorescence microscopy Olympus BX43, equipped with U/B/G filters (Olympus Corporation, Tokyo, Japan). Images were acquired using the Infinity Analyze software version 7.1 (Lumenera Corporation, Ottawa, ON, Canada). Observations focused on discs treated with 100 mg·L^−1^ sakuranetin. Infected discs were cleared (ethanol, NaOH, NaClO), stained with aniline blue, and examined after mounting in water.

#### 4.3.2. Evaluation of the Elicitor Potential of Sakuranetin on Acclimatised Plantlet

##### Treatment and Conditions:

This experiment was conducted under the same conditions as those described in Section 4.2.3 using the same plant material. Bion^®^ (acibenzolar-S-methyl, 2 g·L^−1^) was used as a positive control. Leaves (positions 3 and 4 below the apex) were sampled 4 days after treatment in non-inoculated plants, or 3 days post-inoculation following infection 24 h after treatment. Only plants from the elicitor-effect block were used, and samples were immediately frozen in liquid nitrogen. The sampling time was selected to assess the persistence of the elicitor effect several days after treatment [76].

##### Gene Expression Monitoring by RT-qPCR

Total RNA was extracted from leaf powder using PureLink™ Plant RNA Reagent (Invitrogen, Carlsbad, CA, USA) and reverse transcribed into cDNA. Expression of five defense-related genes (*VvSTS1, VvPR1, VvCHS, VvCHI* and *VvPOX4*) was quantified by RT-qPCR using *VvEF1a, VvUBE2* and *VvACT7* as reference genes. Each biological replicate was analyzed in triplicate. RT-qPCR was performed using SYBR Green chemistry, and relative expression levels were calculated using the ΔΔCq method after normalization.

### 4.4. Statistics

Statistical analyses were performed using GraphPad Prism 10 version 10.6.0 (GraphPad Software, San Diego, CA, USA). Data were tested for normality, and appropriate parametric or non-parametric tests were applied. *P. viticola in vitro* data were analyzed using the Kruskal–Wallis test, whereas *B. cinerea* data were analyzed by one-way ANOVA followed by Tukey’s post hoc test. Zoospore release data were analyzed using the Šidák multiple-comparison test, *P. viticola* infection *in planta* using the Kruskal–Wallis test, and gene expression data by one-way ANOVA followed by Dunnett’s test versus the control. Differences were considered statistically significant at *p* < 0.05.

IC50 values were estimated using a four-parameter logistic (4PL) regression model fitted to log10-transformed concentrations:

$$Y=Bottom+\frac{Top-Bottom}{1+10^{(log_{10}(IC_{50})-X)\times HillSlope}}$$

where Y is the response and X is the log10 inhibitor concentration. IC50 values and their 95% confidence intervals were derived from the fitted model. The standard deviation of IC50 was estimated by error propagation from the log-transformed scale:

$$SD_{log_{10}(IC_{50})}=\frac{CI_{95\%}^{upper}-CI_{95\%}^{lower}}{2\times 1.96}$$

This approach ensures consistency with the non-linear regression model while expressing variability on the linear IC50 scale. Zoospore release data were analyzed using the Šidák multiple-comparison test, *P. viticola* infection *in planta* using the Kruskal–Wallis test, and gene expression data by ANOVA followed by Dunnett’s test versus the control. Differences were considered significant at *p* < 0.05.

## 5. Conclusions

Overall, this study highlights the biological potential of sakuranetin purified from cherry branches, a widely available arboricultural by-product, for the management of *B. cinerea* and *P. viticola* in grapevine. Sakuranetin exhibited strong *in vitro* activity against both pathogens, with IC_50_ values among the lowest reported for natural flavonoids. Its pronounced anti-germinative effect, particularly on *P. viticola* zoospores, suggests that it targets early stages of the pathogen infection cycle. However, this efficacy was not confirmed *in planta* under the tested conditions, as no significant protective effect was observed at the applied concentration. This discrepancy may have resulted from several factors, including limited compound availability at the site of action or the absence of an appropriate formulation. In addition, sakuranetin treatment was associated with the modulation of specific defense-related responses in grapevine. While these observations suggest a potential role in defense activation, our results do not allow us to conclusively demonstrate a biologically effective elicitor activity under the conditions tested. Future studies will aim to elucidate the reasons underlying the lack of *in planta* efficacy. Several hypotheses will be explored, including the determination of optimal application concentrations and the development of improved formulations to enhance sakuranetin stability, solubility, and bioavailability, with the objective of increasing its effectiveness under *in planta* conditions.

## Data Availability

The data presented in this study are available upon request from the corresponding author. They are not publicly accessible due to confidentiality agreements, ongoing research conducted in collaboration with an industrial partner, as well as potential future publications.

## Supplementary Materials

The following supporting information can be downloaded at https://www.mdpi.com/article/10.3390/molecules31081368/s1: Figure S1. ^1^H NMR spectra of the sakuranetin standard and purified sakuranetin; Figure S2. ^13^C NMR spectra of the sakuranetin standard and purified sakuranetin; Figure S3. 2D ^1^H–^1^H COSY NMR spectra of the sakuranetin standard and purified sakuranetin; Figure S4. 2D ^1^H–^13^C HMBC NMR spectra of the sakuranetin standard and purified sakuranetin; Figure S5. 2D ^1^H–^13^C HSQC NMR spectra of the sakuranetin standard and purified sakuranetin; Figure S6. DEPT-135 NMR spectrum of purified sakuranetin; Figure S7. Disease severity scoring scale for infected grape berries; Figure S8. Image processing and analysis workflow for the quantification of foliar infection by *Plasmopara viticola*; Pages S8–S15. Detailed Materials and Methods.

## References

[1] Venkitasamy C., Zhao L., Zhang R., Pan Z. (2019). Grapes. Integrated Processing Technologies for Food and Agricultural By-Products.

[2] The International Organisation of Vine and Wine (OIV) (2025). State of the World Vine and Wine Sector in 2024.

[3] Martelli G. (1997). Infectious diseases and certification of grapevine. Options Mediterr. Ser. B.

[4] Li Z., Wu R., Guo F., Wang Y., Nick P., Wang X. (2025). Advances in the molecular mechanism of grapevine resistance to fungal diseases. Mol. Hortic..

[5] Armijo G., Schlechter R., Agurto M., Muñoz D., Nuñez C., Arce-Johnson P. (2016). Grapevine pathogenic microorganisms: Understanding infection strategies and host response scenarios. Front. Plant Sci..

[6] Koledenkova K., Esmaeel Q., Jacquard C., Nowak J., Clément C., Ait Barka E. (2022). *Plasmopara viticola* the causal agent of downy mildew of grapevine: From its taxonomy to disease management. Front. Microbiol..

[7] Fedorina J., Tikhonova N., Ukhatova Y., Ivanov R., Khlestkina E. (2022). Grapevine Gene Systems for Resistance to Gray Mold *Botrytis cinerea* and Powdery Mildew *Erysiphe necator*. Agronomy.

[8] Zubrod J.P., Bundschuh M., Arts G., Brühl C.A., Imfeld G., Knäbel A., Payraudeau S., Rasmussen J.J., Rohr J., Scharmüller A. (2019). Fungicides: An overlooked pesticide class?. Environ. Sci. Technol..

[9] Landrigan P.J., Fuller R., Acosta N.J., Adeyi O., Arnold R., Baldé A.B., Bertollini R., Bose-O’Reilly S., Boufford J.I., Breysse P.N. (2018). The Lancet Commission on pollution and health. Lancet.

[10] Shekhar C., Khosya R., Thakur K., Mahajan D., Kumar R., Kumar S., Sharma A.K. (2024). A systematic review of pesticide exposure, associated risks, and long-term human health impacts. Toxicol. Rep..

[11] Zhou W., Li M., Achal V. (2025). A comprehensive review on environmental and human health impacts of chemical pesticide usage. Emerg. Contam..

[12] Hawkins N.J., Fraaije B.A. (2016). Predicting resistance by mutagenesis: Lessons from 45 years of MBC resistance. Front. Microbiol..

[13] Jacometti M., Wratten S., Walter M. (2010). Alternatives to synthetic fungicides for *Botrytis cinerea* management in vineyards. Aust. J. Grape Wine Res..

[14] Bavaresco L., Squeri C., Vercesi A. (2019). Field evaluation of new plant protection products against *Plasmopara viticola*. BIO Web Conf..

[15] Gabaston J., Richard T., Cluzet S., Palos Pinto A., Dufour M.-C., Corio-Costet M.-F., Mérillon J.-M. (2017). Pinus pinaster Knot: A Source of Polyphenols against *Plasmopara viticola*. J. Agric. Food Chem..

[16] Marchand P.A. (2023). Evolution of plant protection active substances in Europe: The disappearance of chemicals in favour of biocontrol agents. Environ. Sci. Pollut. Res..

[17] Schneider K., Barreiro-Hurle J., Rodriguez-Cerezo E. (2023). Pesticide reduction amidst food and feed security concerns in Europe. Nat. Food.

[18] Tudi M., Daniel Ruan H., Wang L., Lyu J., Sadler R., Connell D., Chu C., Phung D.T. (2021). Agriculture development, pesticide application and its impact on the environment. Int. J. Environ. Res. Public Health.

[19] Stenberg J.A., Sundh I., Becher P.G., Björkman C., Dubey M., Egan P.A., Friberg H., Gil J.F., Jensen D.F., Jonsson M. (2021). When is it biological control? A framework of definitions, mechanisms, and classifications. J. Pest Sci..

[20] Biocontrol Manufacturers Association What is Biocontrol?. https://ibma-global.org/what-is-biocontrol.

[21] Akula R., Ravishankar G.A. (2011). Influence of abiotic stress signals on secondary metabolites in plants. Plant Signal. Behav..

[22] Castillo F., Aguilar C., Hernández D., Gallegos G., Rodríguez R. (2012). Antifungal Properties of Bioactive Compounds from Plants.

[23] Rejeb I.B., Pastor V., Mauch-Mani B. (2014). Plant Responses to Simultaneous Biotic and Abiotic Stress: Molecular Mechanisms. Plants.

[24] Wink M. (2018). Plant secondary metabolites modulate insect behavior-steps toward addiction?. Front. Physiol..

[25] Liu X., Cao A., Yan D., Ouyang C., Wang Q., Li Y. (2021). Overview of mechanisms and uses of biopesticides. Int. J. Pest Manag..

[26] Harborne J.B., Williams C.A. (2000). Advances in flavonoid research since 1992. Phytochemistry.

[27] Wang Y., Chen S., Yu O. (2011). Metabolic engineering of flavonoids in plants and microorganisms. Appl. Microbiol. Biotechnol..

[28] Al Aboody M.S., Mickymaray S. (2020). Anti-fungal efficacy and mechanisms of flavonoids. Antibiotics.

[29] Liu Y., Fernie A.R., Tohge T. (2022). Diversification of chemical structures of methoxylated flavonoids and genes encoding flavonoid-O-methyltransferases. Plants.

[30] Ibrahim R.K., Bruneau A., Bantignies B. (1998). Plant O-methyltransferases: Molecular analysis, common signature and classification. Plant Mol. Biol..

[31] Stompor M. (2020). A Review on Sources and Pharmacological Aspects of Sakuranetin. Nutrients.

[32] Kodama O., Miyakawa J., Akatsuka T., Kiyosawa S. (1992). Sakuranetin, a flavanone phytoalexin from ultraviolet-irradiated rice leaves. Phytochemistry.

[33] Valletta A., Iozia L.M., Fattorini L., Leonelli F. (2023). Rice phytoalexins: Half a century of amazing discoveries; part I: Distribution, biosynthesis, chemical synthesis, and biological activities. Plants.

[34] Park H.L., Yoo Y., Hahn T.-R., Bhoo S.H., Lee S.-W., Cho M.-H. (2014). Antimicrobial Activity of UV-Induced Phenylamides from Rice Leaves. Molecules.

[35] Yang J., Lai J., Kong W., Li S. (2022). Asymmetric synthesis of sakuranetin-relevant flavanones for the identification of new chiral antifungal leads. J. Agric. Food Chem..

[36] Su M., Han Z., Liu Y., Liu M., Guo L., Wu J., Wu X. (2025). Transcriptome and Metabolome Analyses of Short-Term Responses of *Populus talassica* × *Populus euphratica* to Leaf Damage. Int. J. Mol. Sci..

[37] Huang Z., Hashida K., Makino R., Ohara S., Amartey S., Gillah P. (2010). Flavonoids with antifungal activity from heartwood of Tanzanian wood species: *Commiphora mollis* (Burseraceae). Int. Wood Prod. J..

[38] Hosseinihashemi S.K., Salem M.Z., HosseinAshrafi S.K., Latibari A.J. (2016). Chemical composition and antioxidant activity of extract from the wood of *Fagus orientalis*: Water resistance and decay resistance against *Trametes versicolor*. BioResources.

[39] Hasegawa M., Mitsuhara I., Seo S., Okada K., Yamane H., Iwai T., Ohashi Y. (2014). Analysis on Blast Fungus-Responsive Characters of a Flavonoid Phytoalexin Sakuranetin; Accumulation in Infected Rice Leaves, Antifungal Activity and Detoxification by Fungus. Molecules.

[40] Cho M.-H., Lee S.-W. (2015). Phenolic phytoalexins in rice: Biological functions and biosynthesis. Int. J. Mol. Sci..

[41] Zhang Y., Hu J., Li L., Zhang X., Chen L., Zhou Z., Wang J., Sheng Q., Liang Z., Hong G. (2024). Single-repeat MYB transcription factor, OsMYB1R, enhanced phytoalexin sakuranetin accumulation and Magnaporthe oryzae resistance. Curr. Plant Biol..

[42] Jiang L., Zhang X., Zhao Y., Zhu H., Fu Q., Lu X., Huang W., Yang X., Zhou X., Wu L. (2024). Phytoalexin sakuranetin attenuates endocytosis and enhances resistance to rice blast. Nat. Commun..

[43] Ma J., Wei L., Huang K., Wang D., Gao J., Chen X., Guo H., Gao S., Zhang M., Li S. (2025). Biosynthesis of sakuranetin regulated by O s MPK 6-O s WRKY 67-O s NOMT cascade enhances resistance to false smut disease. New Phytol..

[44] Zhao Y., Zhang X., Lang Z., Zhang C., Li L., He Y., Liu N., Zhu Y., Hong G. (2024). Comparison of Nutritional Diversity in Five Fresh Legumes Using Flavonoids Metabolomics and Postharvest *Botrytis cinerea* Defense Analysis of Peas Mediated by Sakuranetin. J. Agric. Food Chem..

[45] Zhu J., Tang X., Sun Y., Li Y., Wang Y., Jiang Y., Shao H., Yong B., Li H., Tao X. (2022). Comparative metabolomic profiling of compatible and incompatible interactions between potato and *Phytophthora infestans*. Front. Microbiol..

[46] Hu J., Li L., He Y., Hong G., Zhang C. (2024). Searching for the Virulence-contributing Genes of the *Magnaporthe oryzae* by Transcriptome Analysis. Pathogens.

[47] Asahina Y. (1908). Ueber das Sakuranin, ein neues Glykosid der Rinde von Prunus Pseudo-Cerasus Lindl. var. Sieboldi Maxim. Arch. Der Pharm..

[48] Aires A., Dias C., Carvalho R., Saavedra M. (2017). Analysis of glycosylated flavonoids extracted from sweet-cherry stems, as antibacterial agents against pathogenic *Escherichia coli* isolates. Acta Biochim. Pol..

[49] Chaves J.O., De Souza M.C., Da Silva L.C., Lachos-Perez D., Torres-Mayanga P.C., Machado A.P.d.F., Forster-Carneiro T., Vázquez-Espinosa M., González-de-Peredo A.V., Barbero G.F. (2020). Extraction of flavonoids from natural sources using modern techniques. Front. Chem..

[50] Le Bourvellec C., Bureau S., Renard C.M.G.C., Plenet D., Gautier H., Touloumet L., Girard T., Simon S. (2015). Cultivar and Year Rather than Agricultural Practices Affect Primary and Secondary Metabolites in Apple Fruit. PLoS ONE.

[51] Solar A., Medic A., Slatnar A., Mikulic-Petkovsek M., Botta R., Rovira M., Sarraquigne J.-P., Silva A.P., Veberic R., Stampar F. (2022). The Effects of the Cultivar and Environment on the Phenolic Contents of Hazelnut Kernels. Plants.

[52] Gabaston J., Cantos-Villar E., Biais B., Waffo-Teguo P., Renouf E., Corio-Costet M.-F., Richard T., Mérillon J.-M. (2017). Stilbenes from *Vitis vinifera* L. Waste: A Sustainable Tool for Controlling *Plasmopara Viticola*. J. Agric. Food Chem..

[53] Pezet R., Gindro K., Viret O., Richter H. (2004). Effects of resveratrol, viniferins and pterostilbene on *Plasmopara viticola* zoospore mobility and disease development. Vitis J. Grapevine Res..

[54] Harm A., Kassemeyer H.-H., Seibicke T., Regner F. (2011). Evaluation of chemical and natural resistance inducers against downy mildew (*Plasmopara viticola*) in grapevine. Am. J. Enol. Vitic..

[55] Zorrilla J.G., Giovannini O., Nadalini S., Zanini A., Russo M.T., Masi M., Puopolo G., Cimmino A. (2024). Suppressive Activity of Glechoma hederacea Extracts against the Phytopathogenic Oomycete *Plasmopara viticola*, and First Screening of the Active Metabolites. Agriculture.

[56] Thuerig B., Ramseyer J., Hamburger M., Oberhänsli T., Potterat O., Schärer H.J., Tamm L. (2016). Efficacy of a Juncus effusus extract on grapevine and apple plants against *Plasmopara viticola* and *Venturia inaequalis*, and identification of the major active constituent. Pest Manag. Sci..

[57] Thuerig B., James E.E., Schärer H.J., Langat M.K., Mulholland D.A., Treutwein J., Kleeberg I., Ludwig M., Jayarajah P., Giovannini O. (2018). Reducing copper use in the environment: The use of larixol and larixyl acetate to treat downy mildew caused by *Plasmopara viticola* in viticulture. Pest Manag. Sci..

[58] Zhang J., Ma S., Du S., Chen S., Sun H. (2019). Antifungal activity of thymol and carvacrol against postharvest pathogens *Botrytis cinerea*. J. Food Sci. Technol..

[59] Wang C., Zhang J., Chen H., Fan Y., Shi Z. (2010). Antifungal activity of eugenol against *Botrytis cinerea*. Trop. Plant Pathol..

[60] El Khawand T., Gabaston J., Taillis D., Iglesias M.-L., Pedrot E., Pinto A.P., Fonayet J.V., Merillon J.M., Decendit A., Cluzet S. (2020). A dimeric stilbene extract produced by oxidative coupling of resveratrol active against *Plasmopara viticola* and *Botrytis cinerea* for vine treatments: This article is published in cooperation with the 11th OenoIVAS International Symposium, June 25–28 2019, Bordeaux, France. OENO One.

[61] Cotoras M., Mendoza L., Muñoz A., Yáñez K., Castro P., Aguirre M. (2011). Fungitoxicity against *Botrytis cinerea* of a flavonoid isolated from *Pseudognaphalium robustum*. Molecules.

[62] Santier S., Chamel A. (1998). Reassessment of the role of cuticular waxes in the transfer of organic molecules through plant cuticles. Plant Physiol. Biochem..

[63] Schreiber L. (2006). Review of sorption and diffusion of lipophilic molecules in cuticular waxes and the effects of accelerators on solute mobilities. J. Exp. Bot..

[64] Chaaban H., Ioannou I., Paris C., Charbonnel C., Ghoul M. (2017). The photostability of flavanones, flavonols and flavones and evolution of their antioxidant activity. J. Photochem. Photobiol. A Chem..

[65] Phong T.K., Nhung D.T.T., Yamazaki K., Takagi K., Watanabe H. (2008). Simulated rainfall removal of tricyclazole sprayed on rice foliage. Bull. Environ. Contam. Toxicol..

[66] Atkinson P., Blakeman J.P. (1982). Seasonal Occurrence of an Antimicrobial Flavanone, Sakuranetin, Associated with Glands on Leaves of *Ribes nigrum*. New Phytol..

[67] Katsumata S., Hamana K., Horie K., Toshima H., Hasegawa M. (2017). Identification of sternbin and naringenin as detoxified metabolites from the rice flavanone phytoalexin sakuranetin by *Pyricularia oryzae*. Chem. Biodivers..

[68] Katsumata S., Toshima H., Hasegawa M. (2018). Xylosylated Detoxification of the Rice Flavonoid Phytoalexin Sakuranetin by the Rice Sheath Blight Fungus Rhizoctonia solani. Molecules.

[69] Schouten A., Wagemakers L., Stefanato F.L., van der Kaaij R.M., van Kan J.A. (2002). Resveratrol acts as a natural profungicide and induces self-intoxication by a specific laccase. Mol. Microbiol..

[70] Pedras M.S.C., Ahiahonu P.W. (2005). Metabolism and detoxification of phytoalexins and analogs by phytopathogenic fungi. Phytochemistry.

[71] Kraus C., Abou-Ammar R., Schubert A., Fischer M. (2021). *Warburgia ugandensis* Leaf and Bark Extracts: An Alternative to Copper as Fungicide against Downy Mildew in Organic Viticulture?. Plants.

[72] Ozfidan-Konakci C., Yildiztugay E., Alp F.N., Kucukoduk M., Turkan I. (2020). Naringenin induces tolerance to salt/osmotic stress through the regulation of nitrogen metabolism, cellular redox and ROS scavenging capacity in bean plants. Plant Physiol. Biochem..

[73] Willig G., Brunissen F., Brunois F., Godon B., Magro C., Monteux C., Peyrot C., Ioannou I. (2022). Phenolic Compounds Extracted from Cherry Tree (*Prunus avium*) Branches: Impact of the Process on Cosmetic Properties. Antioxidants.

[74] Peressotti E., Duchêne E., Merdinoglu D., Mestre P. (2011). A semi-automatic non-destructive method to quantify grapevine downy mildew sporulation. J. Microbiol. Methods.

[75] Holz G., Coertze S., Williamson B., Elad Y., Williamson B., Tudzynski P., Delen N. (2007). The Ecology of Botrytis on Plant Surfaces. Botrytis: Biology, Pathology and Control.

[76] Burdziej A., Bellée A., Bodin E., Valls Fonayet J., Magnin N., Szakiel A., Richard T., Cluzet S., Corio-Costet M.-F. (2021). Three Types of Elicitors Induce Grapevine Resistance against Downy Mildew via Common and Specific Immune Responses. J. Agric. Food Chem..

