# Supplementary material for: Sakuranetin, a Natural Flavonoid, Promising to Manage Grapevine Diseases
Source: Molecules. 2026 Apr 21;31(8):1368. doi: 10.3390/molecules31081368 (PMC13118415; doi:10.3390/molecules31081368)
Supplement: Supplementary file 1 [file molecules-31-01368-s001.zip › molecules-4250340-supplementary.pdf]

Figure ***S1 :*** ***^{1}H NMR spectra of the sakuranetin standard and purified sakuranetin***

Standard of sakuranetin

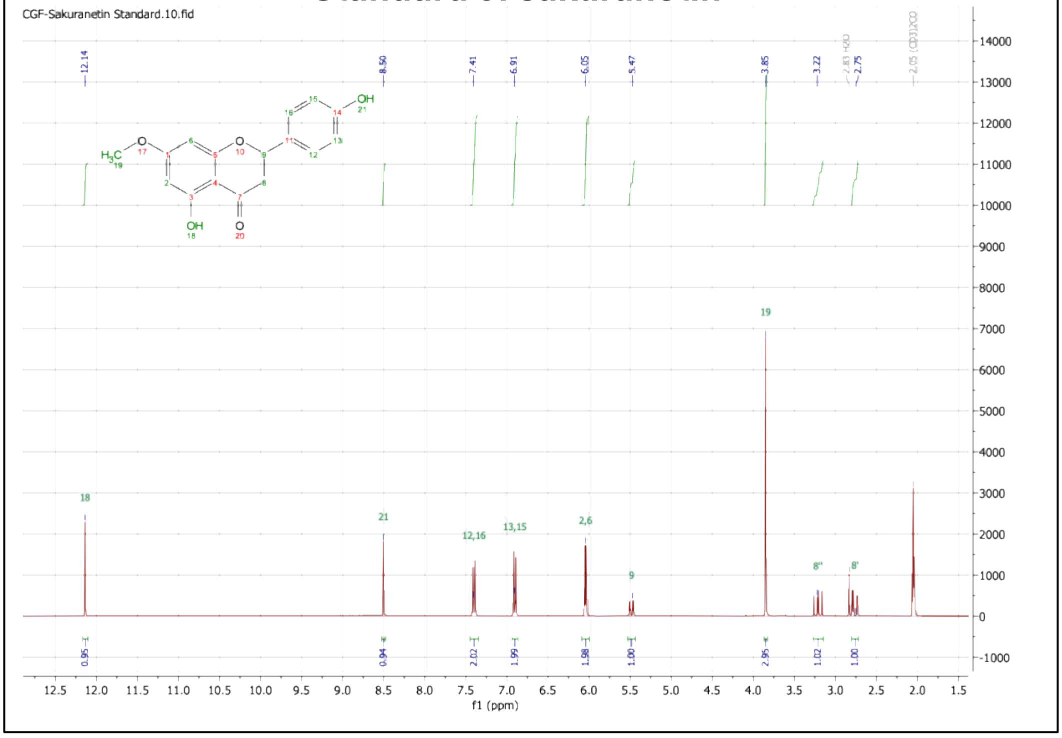

Purified sakuranetin

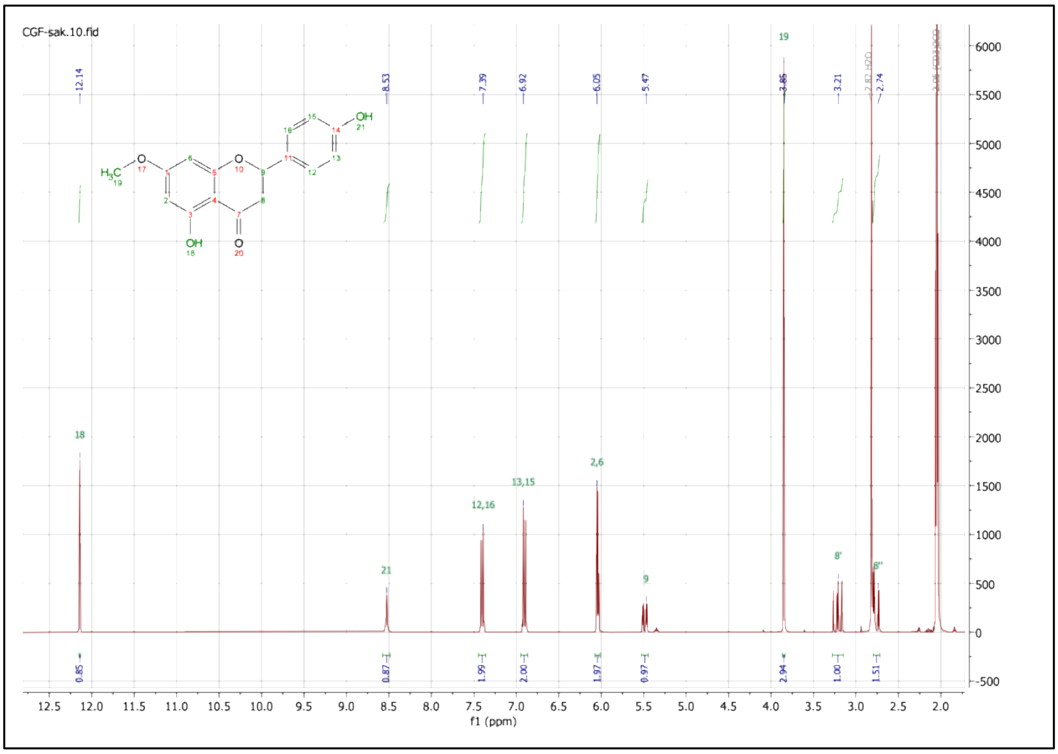

^{1}H NMR (300 MHz, Acetone-d_6) δ (ppm) 2.74 (dd, *J* = 17.17 Hz, *J* = 3.08 Hz, **1H**), 3.21 (dd, *J* = 17.17 Hz, *J* = 12.75 Hz, **1H**), 3.85 (s, **3H**), 5.47 (dd, *J* = 12.74 Hz, *J* = 3.00 Hz, **1H**), 6.04 (d, *J* = 2.26 Hz, **1H**), 6.05 (d, *J* = 2.22 Hz, **1H**), 6.90 (m, **1H**), 6.92 (m, **1H**), 7.39 (m, **1H**), 7.41 (m, **1H**), 8.53 (s, **1H**), 12.14 (s, **1H**).

Figure ***S2 :*** ***^{13}C NMR spectra of the sakuranetin standard and purified sakuranetin***

Standard of sakuranetin

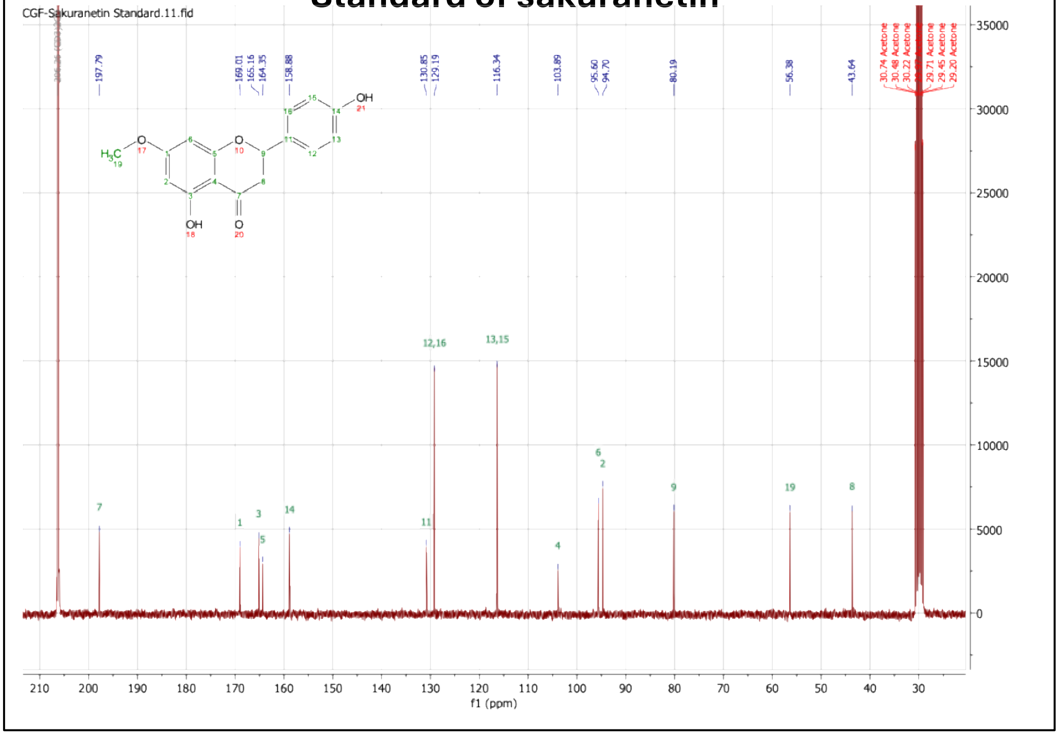

Purified sakuranetin

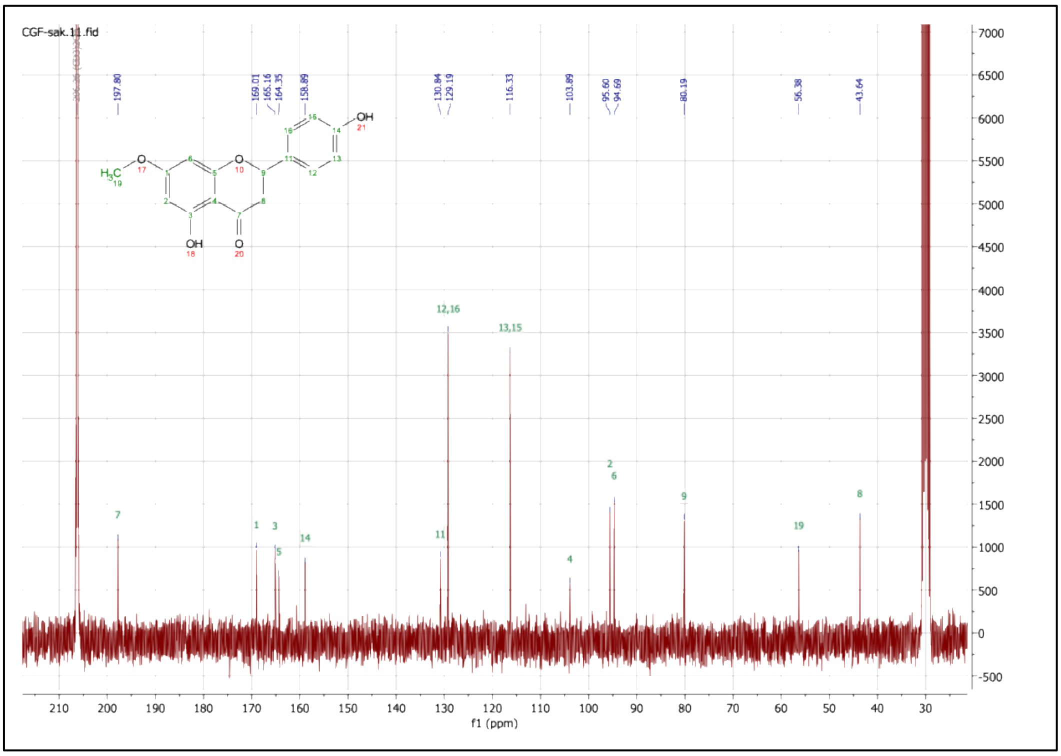

^{13}**CNMR** (75 MHz, Acetone-d_6) δ (ppm) 43.6 (**CH_2**), 56.4 (**CH_3**), 80.2 (**CH**), 94.7 (**CH**), 95.6 (**CH**), 103.9 (**C**), 116.3 (2x**CH**), 129.2 (2x**CH**), 130.9 (**C**), 158.9 (**C**), 164.3 (**C**), 165.2 (**C**), 169.0 (**C**), 197.8 (**C**).

Figure S3: 2D ^{1}H–^{1}H COSY NMR spectra of the sakuranetin standard and purified

Standard of sakuranetin

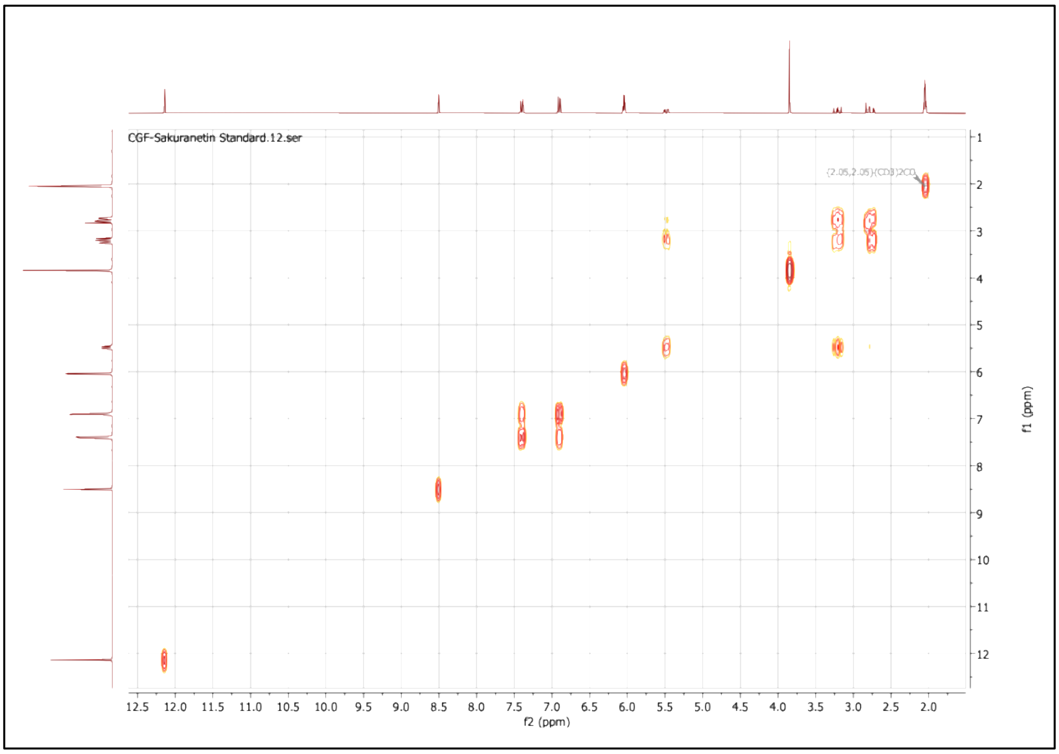

Purified sakuranetin

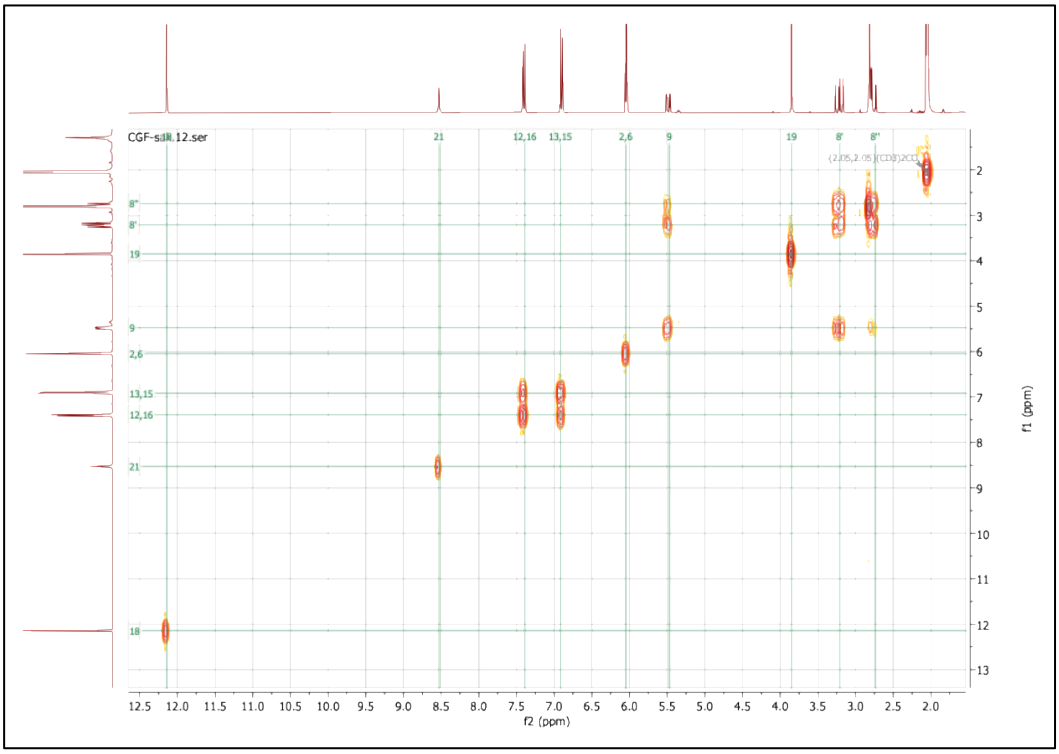

Figure S4: 2D $^{1}H-^{13}C$ HMBC NMR spectra of the sakuranetin standard and purified

Standard of sakuranetin

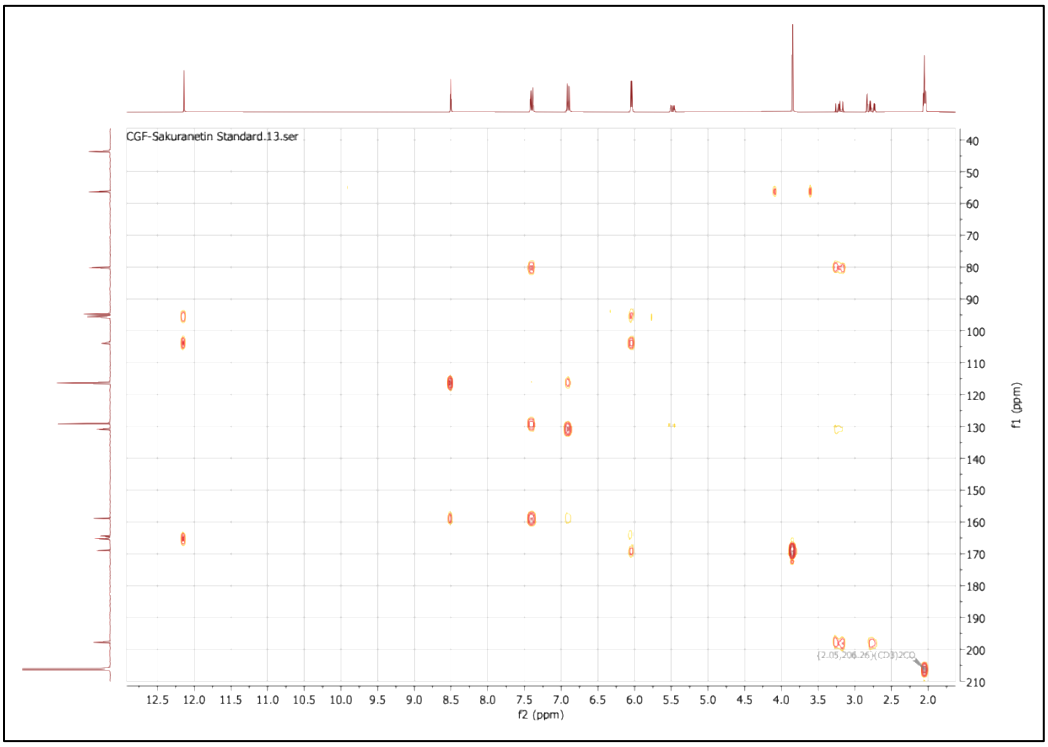

Purified sakuranetin

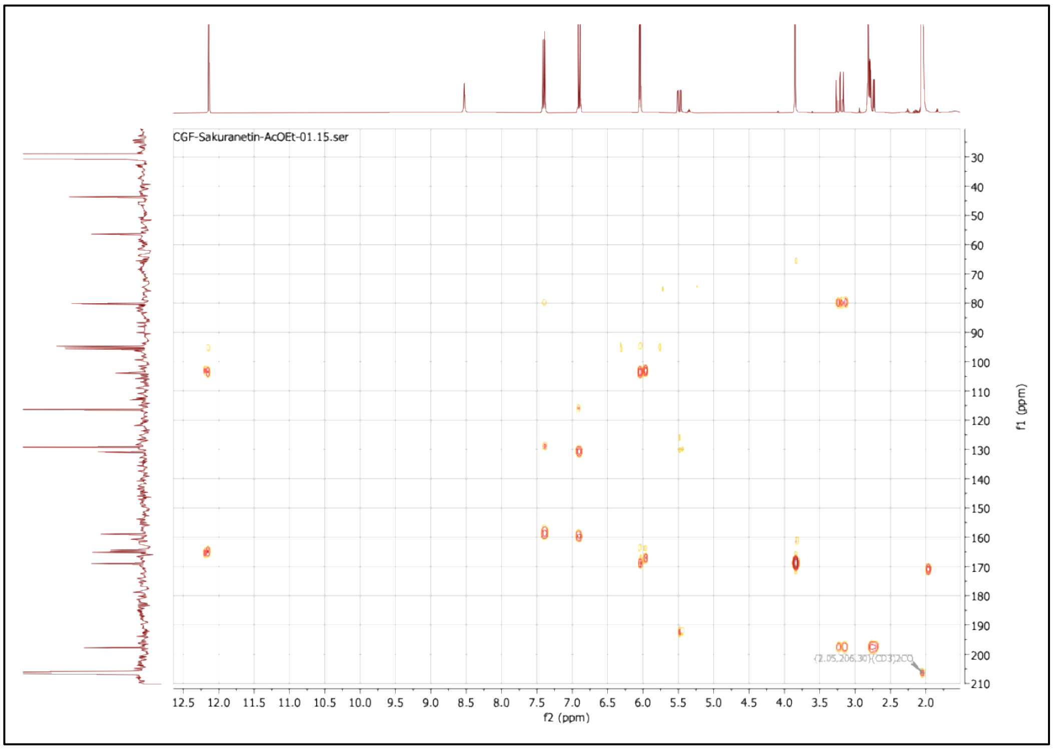

Figure S5: 2D ^1H – ^{13}C HSQC NMR spectra of the sakuranetin standard and purified

Standard of sakuranetin

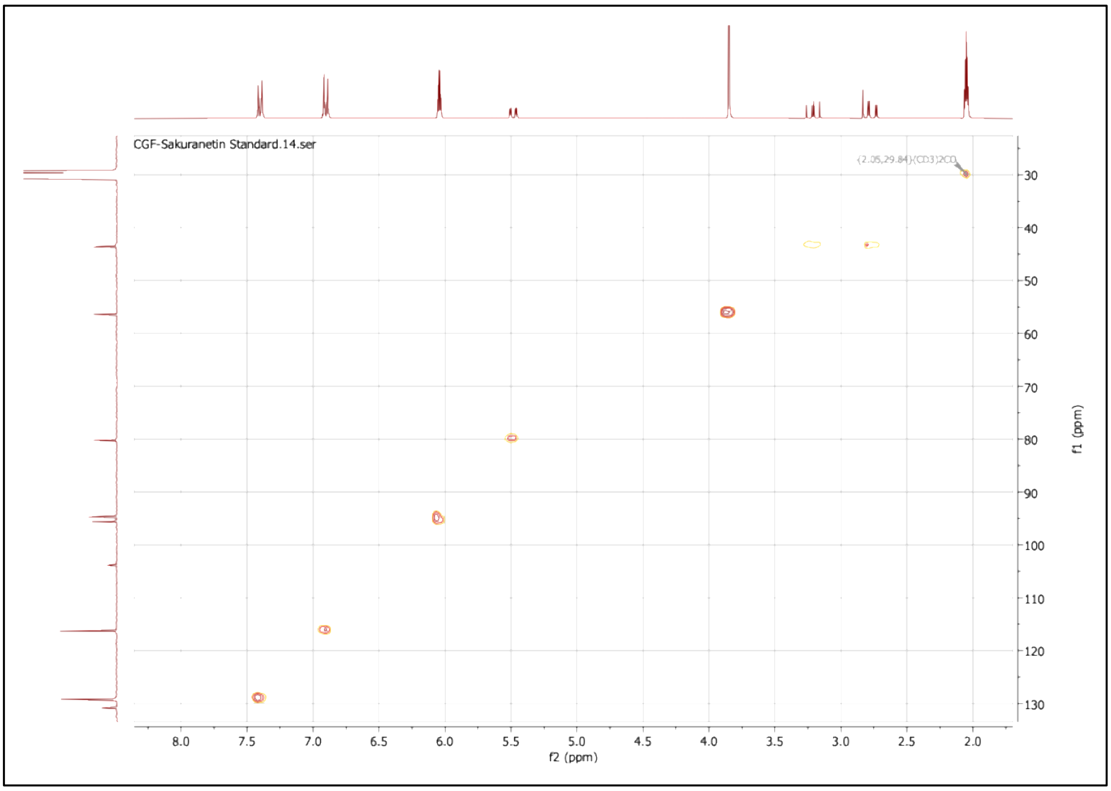

Purified sakuranetin

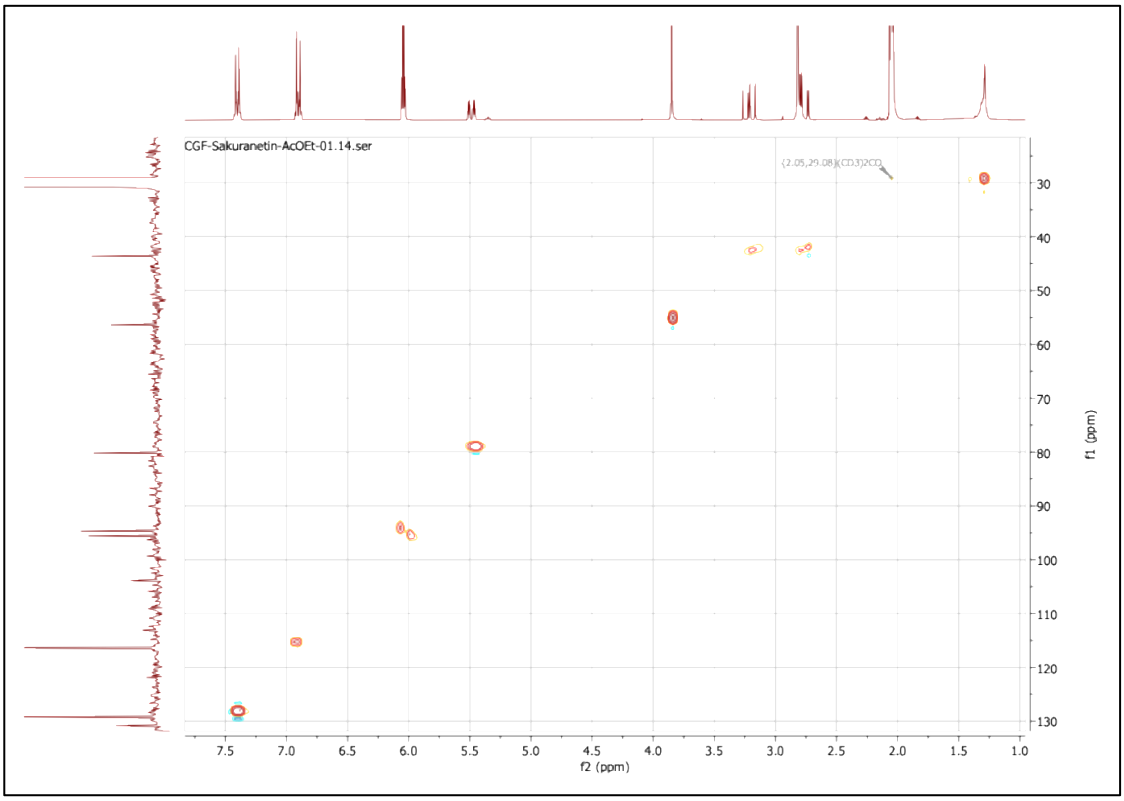

Figure ***S6: DEPT-135 NMR spectra of the purified sakuranetin***

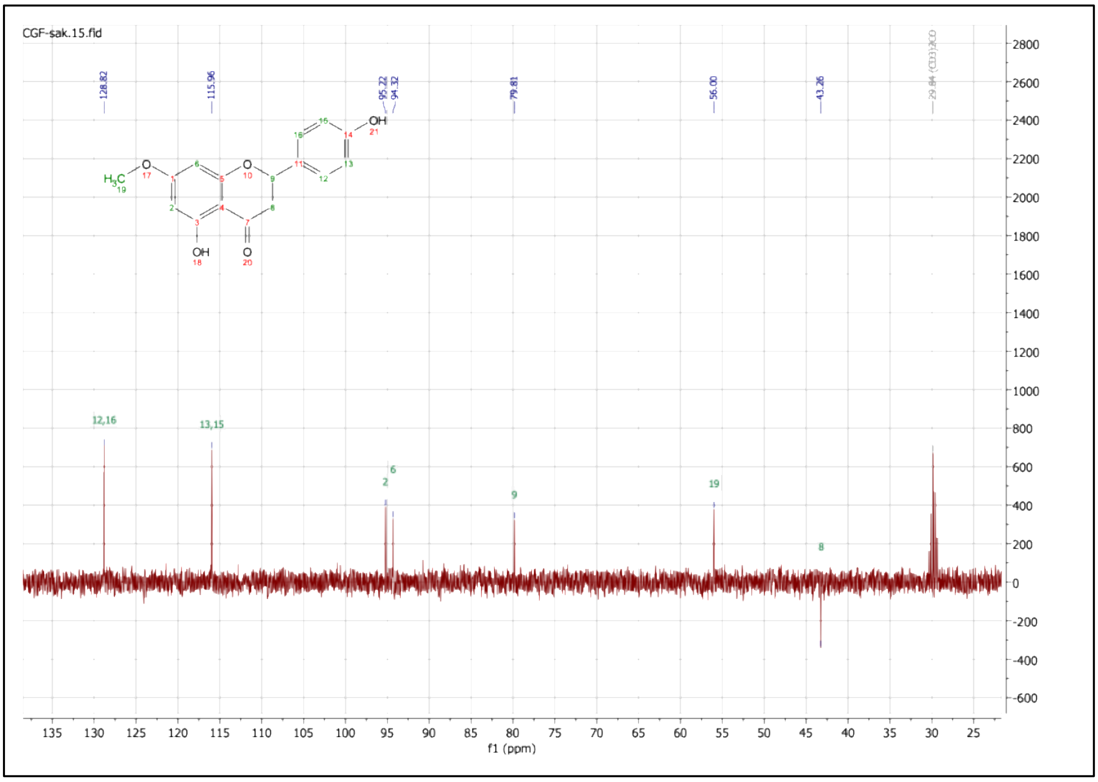

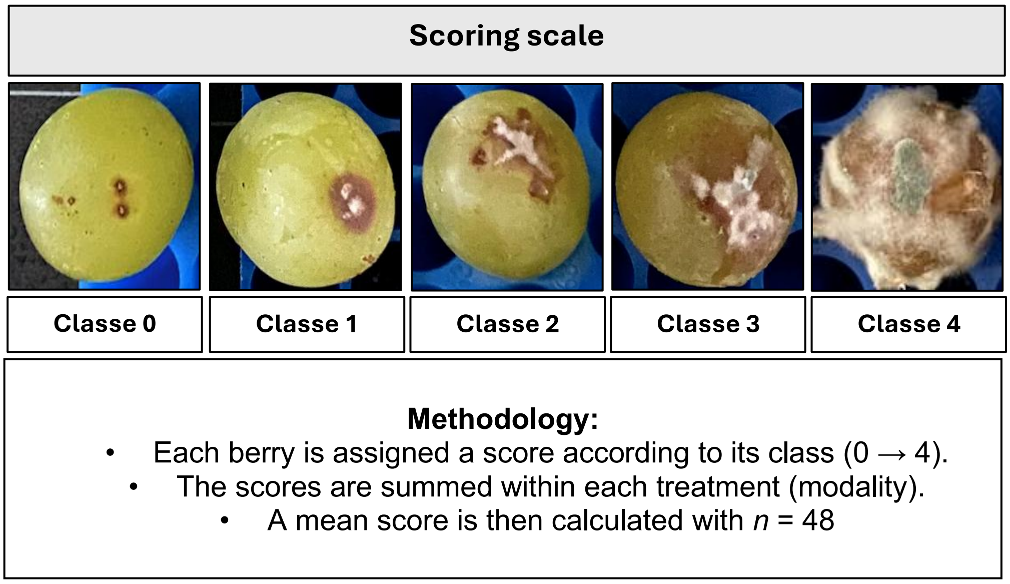

Figure S7 : Disease severity scoring scale for infected grape berries. Representative images of grape berries illustrating the disease severity scoring scale used in this study. Infection severity is classified from Class 0 (no visible symptoms) to Class 4 (severe infection with extensive tissue colonization). This scale was used as a reference to consistently assess and quantify infection levels in grape berries.

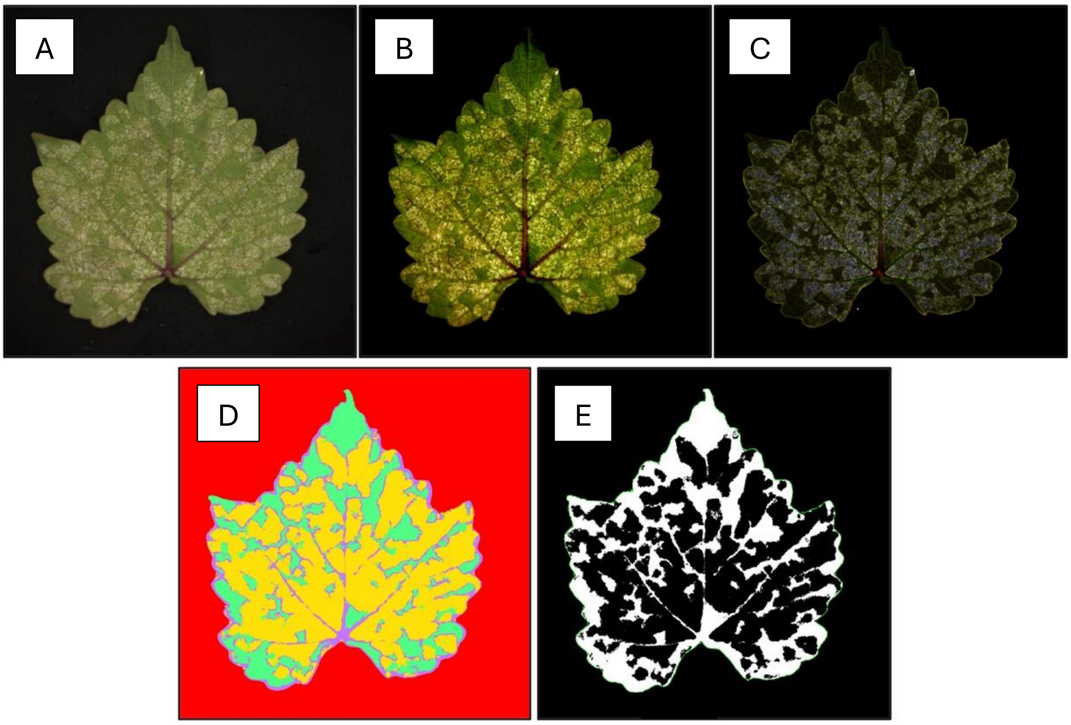

Figure S8 : Image processing and analysis workflow for the quantification of foliar infection by Plasmopara viticola. (A) Raw image of the leaf acquired using a Scan 500 scanner on a non-reflective black background. (B) Contrast enhancement to improve the visibility of infected areas. (C) Application of the “Gradient” filter from the MorphoLibJ plugin to highlight edges and sharp intensity transitions. (D) Supervised segmentation using the Trainable Weka Segmentation (TWS) plugin to generate a binary image separating infected and healthy tissues. (E) Thresholding applied within a region of interest (ROI) corresponding to the leaf area (outlined in green); sporulating areas are shown in black, while non-infected tissues appear in white.

S4. Materials and Methods

S4.1. Obtaining Sakuranetin from Plant Material

S4.1.1. Extraction and Preparation of the Crude Extract from Cherry Tree Branches:

Cherry branches (*Prunus avium* var. *burlat*) were collected in south-eastern France (Drôme department, Chestnut site, Montélimar, 44°33'31" N, 04°45'03" E). Sample preparation and extraction followed [73] with minor modifications. Branches were ground using an SM300 knife mill (Retsch, Germany; 4 mm sieve, 1500 rpm, 1–2 min) and the powder was stored at 4 °C. Extraction was performed by reflux maceration in ethanol/water (70/30, v/v) by mixing 1000 mg of powder with 50 mL of solvent and heating at 70 °C for 30 min under stirring. After cooling, the extract was filtered, centrifuged (4000 × g, 10 min, 25 °C), vacuum-filtered (5 µm), concentrated under reduced pressure and freeze-dried, yielding approximately 75 g of crude extract.

S4.1.2. Detection and Identification of Sakuranetin in Crude Cherry Branch Extract:

LC–MS analyses were performed using an Agilent 1290 UPLC system coupled to a 6545 Q-ToF mass spectrometer equipped with a UV-DAD detector (Agilent Technologies, Santa Clara, CA, USA). Separation was achieved on a Zorbax Eclipse Plus C18 column (50 × 2.1 mm, 1.8 µm) maintained at 40 °C, with a flow rate of 0.4 $mL·min^{-1}$ and an injection volume of 1 µL. The mobile phase consisted of solvent A (H_2O + 0.1% formic acid, v/v) and solvent B (acetonitrile + 0.1% formic acid, v/v). The elution gradient was: 0–0.5 min, 5% B; 0.5–2 min, 5–7% B; 2–6 min, 7–8% B; 6–7.5 min, 8–15% B; 7.5–12 min, 15–25% B; 12–15.5 min, 25–28% B; 15.5–17 min, 28–45% B; 17–18.5 min, 45–65% B; 18.5–19.5 min, 65–85% B; 19.5–20.5 min, 85–100% B; 20.5–23 min, 100% B; 23–23.5 min, 100–5% B; 23.5–24 min, 5% B. UV detection was performed at 210, 254, 285 and 320 nm. MS analyses were conducted in negative ESI mode over an m/z range of 50–1050 at an acquisition rate of 2 $spectra·s^{-1}$. Source parameters were set as follows: capillary voltage 3500 V, nozzle voltage 2000 V, fragmentor 175 V, skimmer 1 at 65 V, octopole 750 V, nebuliser gas at 325 °C (8 $L·min^{-1}$) and sheath gas at 325 °C (11 $L·min^{-1}$). Internal reference ions were m/z 112.985587 and 1033.988109. Data were processed using MassHunter software (v12.0.0) (Agilent Technologies, Santa Clara, CA, USA) and compared with internal, NIST and Metlin databases. Identification of sakuranetin was based on its exact mass (M = 286.0841 Da; $[M–H]^-$ = 285.0763 *m/z*) and confirmed by comparison with a commercial standard, using retention time and MS profiles.

S4.1.3. Two-Step Purifications of Sakuranetin from Crude Cherry Branch Extract:

Step 1 - Fractionation by Flash Chromatography and Identification of the Fraction Containing Sakuranetin:

Preliminary fractionation of the crude extract was performed to reduce sample complexity and enrich sakuranetin prior to preparative HPLC. Fractionation was carried out using a Puriflash PF-5.250-UV800 system (Interchim, Montluçon, France) equipped

with a C18 reverse-phase column (30 µm, 80 g). For each run, 1 g of crude extract was adsorbed onto 10 g of celite, dissolved in ethanol/water (50%, v/v), dried under vacuum, ground and packed into a 12 g dryload cartridge. Elution was performed using solvent A (water) and solvent B (isopropanol), both containing 0.1% acetic acid, with the following gradient: 5 CV 100% A, 5 CV 95:5 A/B, 5 CV 90:10 A/B, 2 CV 85:15 A/B, 5 CV 75:25 A/B and 5 CV 50:50 A/B, including 2 CV transitions between steps, at a flow rate of 50 $mL \cdot min^{-1}$. Fractions were collected based on UV detection at 254 and 280 nm (threshold 10 mAU) and pooled according to their spectral profiles. The fraction containing sakuranetin was identified by UPLC-ESI-QToF-MS analysis as described in Section 2.1.2.

Step 2 - Purification of Sakuranetin by Preparative HPLC from the Fraction of Interest:

Sakuranetin was purified by preparative HPLC using a Waters system (GILoop pump, 2767 autosampler, 2998 PDA detector and SQ Detector 2 MS) (Waters Corporation, Milford, MA, USA). Samples were injected in partial loop mode (700 µL) and separated on a Surf C18 Wax column (250 × 21.2 mm, 5 µm) at 20 $mL \cdot min^{-1}$. The mobile phase consisted of water (A) and methanol (B), both with 0.1% formic acid, using a linear gradient from 100% A to 100% B over 60 min, followed by 20 min at 100% B and column re-equilibration. MS detection was performed in negative ESI mode (*m/z* 50–500), with full-scan acquisition and MassLynx processing, while UV detection was monitored between 190 and 400 nm. Sakuranetin was collected based on combined UV detection and the $[M–H]^-$ ion at *m/z* 285.1.

S4.1.4. Structural Verification of Purified Sakuranetin:

Structural confirmation of purified sakuranetin was performed by NMR spectroscopy to exclude the presence of isomeric compounds. ^{1}H, ^{13}C and two-dimensional NMR experiments (COSY, HSQC, HMBC and DEPT-135) were carried out using a Bruker Ultrashield 300 MHz spectrometer (Bruker, Billerica, MA, USA) equipped with a TXI cryogenic probe. Samples were dissolved in acetone-d_6 and analyzed in 3 mm NMR tubes. Data acquisition was performed using TopSpin software (v3.2) (Bruker, Billerica, MA, USA), while data processing, including integration and peak assignment, was carried out using MestReNova software version 14.0.0-23239 (Mestrelab Research, Santiago de compostela, Spain). Spectra obtained for the purified compound were compared with those of an analytical sakuranetin standard analyzed under identical conditions, confirming structural identity.

S4.1.5. Solvent and Reference Substance:

The sakuranetin analytical standard was purchased from Extrasynthese (Lyon, France) and its identity and purity (≥90%) were confirmed by UV–visible diode array spectroscopy, LC–MS and NMR analyses. Acetonitrile used for UPLC analyses was obtained from VWR (VWR international, Radnor, PA, USA), while formic and acetic acids were supplied by Fisher Scientific (Thermo Fishcer Scientific, Waltham, Massachusetts, USA). Ethanol, methanol and isopropanol used for extraction, fractionation and purification were purchased from VWR. Ultrapure water was produced using a Milli-Q Integral 5 system (Merck Millipore, Burlington, MA, USA).

S4.2. Evaluation of the Protective Activity of Purified Sakuranetin Against Two Major Grapevine Pathogens

S4.2.1. Biological Material:

For *P. viticola*, both *in vitro* and *in planta* experiments were performed using plant material derived from *in vitro*-grown grapevine plantlets (*Vitis vinifera* cv. Chardonnay) after acclimatization. *In vitro* assays were conducted on leaf discs excised from acclimatized plantlets, while *in planta* experiments were carried out on acclimatized plantlets at the 12-leaf stage. Plantlets were grown for 8 weeks on Murashige and Skoog medium (25 °C, 16 h/8 h light/dark), transferred to potting soil, acclimatized for 3 weeks under near-saturated humidity, and then maintained in a conventional greenhouse. Plants were staked and disbudded to promote main shoot growth. Young, non-stressed leaves with visible veins were surface sterilized in 70% ethanol for 30 s and rinsed three times with sterile ultrapure water. *P. viticola* (Arbiotech ARB strain) was maintained on sterilized grapevine leaves on 10% agar. Inoculation was performed by spraying a spore suspension (1×10^4 spores·mL^{-1}) onto the abaxial leaf surface, followed by incubation at 24 °C in the dark for 24 h and then under a 16 h/8 h light/dark photoperiod, with weekly subculturing. *Botrytis cinerea* strain BC630 was cultured on potato dextrose agar (PDA, 39 g·L^{-1}) from glycerol-preserved spore stocks (≈1×10^7 spores·mL^{-1}) and incubated at 20 °C in the dark.

S4.2.2. In Vitro Evaluation of Purified Sakuranetin

For both pathogens**,** Sakuranetin was prepared at concentrations of 0, 1, 5, 10, 25, 50, 75, and 100 mg·L^{-1} in water containing 1% ethanol. The solutions were kept in an ultrasonic bath until use to maintain the solubility of sakuranetin.

Evaluation of Anti-Oomycete Activity Against *Plasmopara viticola*:

Leaf discs (15 mm diameter) were excised from leaves of acclimatized *Vitis vinifera* cv. Chardonnay plantlets and randomly distributed (12 discs per treatment) in Petri dishes containing 10 mL of 10% agar. Sakuranetin solutions were sprayed onto the discs 24 h before inoculation (2 mL per dish) and dried under sterile conditions, while control discs received sterile water with 1% ethanol. Inoculation was carried out by spraying 2 mL per dish of a *P. viticola* spore suspension (1×10^4 spores·mL^{-1}). Dishes were incubated for 24 h in the dark and then for 7 days at 22 °C under a 16 h/8 h light/dark photoperiod (35 µmol·m^{-2}·s^{-1}). Experiments were repeated three times independently. Disease development was quantified using an image thresholding method with Fiji software version 20250529-2217 (ImageJ distribution, National Institutes of Health, Bethesda, MD, USA) as described by [74], and results were expressed as percentage inhibition relative to the control. Thresholding segments images based on pixel intensity or color to identify sporulating areas; however, this approach may underestimate disease severity compared to conventional visual scoring.

Evaluation of Antifungal Activity Against *Botrytis cinerea*:

PDA medium was prepared by dissolving 39 g·L^{-1} of Potato Dextrose Agar (PDA, Difco™, Becton Dickinson, commercial product) powder in ultrapure water and sterilized by autoclaving (121 °C, 20 min). After cooling, sakuranetin stock were added, with a final

ethanol content of 1%. Under sterile conditions, 8 mL of medium was poured into 55 mm Petri dishes, with six replicates per treatment. Mycelial plugs of *B. cinerea* (3 mm diameter) taken from one-week-old cultures were placed at the centre of each dish with the mycelial side facing the agar. Plates were sealed with Parafilm and incubated at 20 °C in the dark for 3 days. Mycelial growth was quantified using Fiji software by comparing the colonized area with that of the control, and results were expressed as percentage inhibition. Experiment was performed independently three times.

S4.2.3. Confirmation of the Anti-*Plasmopara viticola* Activity in Planta of Purified Sakuranetin on Acclimatised Plantlets

Greenhouse Experiment and Inoculation with *Plasmopara viticola*:

The experiment was conducted in an experimental greenhouse under natural light (20–28 °C). Each treatment included six acclimatized grapevine plantlets placed in transparent airtight boxes. The design comprised two blocks: a protection block (18 plants distributed in three boxes) and an elicitor-effect block (36 plants divided into two batches of 18 plants, each distributed in four boxes), with six plants per box. This design was implemented to avoid assessing disease development on plants that had undergone leaf sampling, which could alter plant physiology and potentially influence disease progression. Accordingly, plants from the protection block were used exclusively to quantify downy mildew development under direct protection conditions, whereas plants from the elicitor-effect block were used solely for leaf sampling to assess the elicitor potential of sakuranetin. Treatments were applied preventively 24 h before inoculation using a manual sprayer, with 12 mL of solution per plant (≈1 mL per leaf). *P. viticola* sporangia were prepared as described above and adjusted to 2×10^4 sporangia·mL−1. Inoculation was performed by spraying 12 mL of suspension per plant on both leaf surfaces. Saturated humidity was maintained by spraying water on the inner walls of the boxes every two days. After inoculation, plants were incubated in the dark for 24 h and then returned to normal day/night conditions for a total incubation period of 10 days. For the protection block, three treatments were tested: a negative control (water containing 1% ethanol), a positive control consisting of RSR Disperss® Bordeaux mixture (UPL) applied at 3.75 kg·ha^{-1} (18.75 g·L^{-1} for a spray volume of 200 L·ha^{-1}), and sakuranetin applied at 25 mg·L^{-1} in water containing 1% ethanol. The Bordeaux mixture was selected as a positive control because it is a widely used reference in organic agriculture, particularly in viticulture, where it remains one of the effective treatments available for controlling downy mildew. This concentration of sakuranetin was selected as it corresponded to the onset of the plateau phase in the dose–response relationship and provided approximately 80% efficacy *in vitro* on leaf discs, suggesting its suitability for *in planta* testing.

Evaluation of the Protection, Image Processing and Data Acquisition:

After incubation were harvested, placed in moistened Petri dishes and temporarily stored at 4 °C prior to imaging. The abaxial leaf surface was photographed on a black anti-reflective background using a Scan 500 device. Disease development was quantified with Fiji version 20250529-2217 (ImageJ distribution, National Institutes of Health, Bethesda, MD, USA) using image thresholding based on binarized images generated by the

Trainable Weka Segmentation (TWS) plugin. Images were pre-processed by contrast enhancement and application of a gradient filter (MorphoLibJ plugin) before TWS analysis. The model was trained on 24 representative leaves from the experiment and included four classes: background, leaf tissue, sporulation and veins (Supporting Information, Fig. S8). Quantification of protection was performed exclusively on plants from the protection block. For each plant, all leaves (i.e., 12 leaves per plant) were collected and included in the analysis. Infection was calculated as the proportion of leaf area covered by sporulation relative to the total leaf surface.

S4.2.4. Confirmation of the In Vivo Ant*Botrytis cinerea* Activity of Purified Sakuranetin on Detached Berries

Plant Material/Detached Berries:

Grape bunches of a commercial white table Muscat variety were purchased from a supermarket. Berries were detached with their pedicels, surface-sterilized in 70% ethanol for 1 min, and rinsed three times in sterile Milli-Q water (1 min each) under laminar flow conditions. The berries were placed on Eppendorf-type microtube racks, with pedicels inserted into holes filled with 2 mL of sterile Milli-Q water to maintain basal physiological conditions. Each treatment consisted of 48 berries distributed over two racks (24 berries per rack).

Treatments and Inoculation of *Botrytis cinerea*:

Treatments were applied preventively 24 h before inoculation by spraying 7 mL per rack using a sterile manual sprayer. After treatment, berries were dried under a laminar flow hood and kept there for 24 h before inoculation. All solutions were prepared in water containing 1% ethanol and kept in an ultrasonic bath until use to preserve sakuranetin solubility. A commercial anti-*Botrytis* fungicide, Géxe WG® (Syngenta), containing fludioxonil (500 g·kg^{-1}), was used as a positive control at the recommended dose of 5 g·L^{-1} (1 kg·ha^{-1}), corresponding to 2.5 g·L^{-1} of fludioxonil. Sakuranetin was applied at a concentration of 25 mg·L^{-1}. Berries were artificially wounded prior to inoculation to facilitate infection, as *B. cinerea* frequently infects grape berries through wounds or damaged tissues [75]. Two wounds (3 mm deep) were made at the top of each berry using a sterile syringe, and 20 µL of a *B. cinerea* spore suspension (1 × 10^{6} spores·mL^{-1}) was applied to each wound. Racks were placed in sealed transparent boxes containing 500 mL of water to ensure 100% relative humidity and incubated for 7 days in a culture chamber under a 16 h/8 h light/dark photoperiod.

Quantification and Evaluation of the Protection:

To quantify the protection a rating scale was established to classify each berry according to its degree of infection, thereby enabling an objective comparison of the different modalities (supporting information; fig. S7).

S4.3. Elucidation of Protective Activity Through Mechanistic Study of Purified Sakuranetin

S4.3.1. Microscopic Observation at Different Stages of the *Plasmopara viticola* Infection Cycle

Impact on Sporangia Release, Motility and Spore Germination:

A spore suspension was adjusted to 2 × 10^4 spores·mL^{-1} and 100 µL was dispensed into each well of a 96-well plate. To limit premature zoospore release, the suspension was kept on ice until plate filling was completed. Subsequently, 100 µL of sakuranetin stock solutions were added to obtain final concentrations of 1, 10 and 100 mg·L^{-1}, corresponding to a final spore concentration of 1 × 10^4 spores·mL^{-1} in medium containing 1% ethanol. Each condition was tested in six replicate wells. At time 0 h, no sporangia opening or zoospore release was observed. Observations were performed using an automated optic microscope EVOS M7000 (Thermo Fisher Scientific, Waltham, Massachusetts, USA) at ×20 magnification. One image per well was acquired at 0, 3 and 6 h after treatment. Image analysis was carried out using Fiji software version 20250529-2217 (ImageJ distribution, National Institutes of Health, Bethesda, MD, USA). Zoospore release was quantified by calculating the ratio of empty sporangia to the total number of sporangia, expressed as a percentage. Zoospore germination was defined by the appearance of a germ tube but, due to difficulties in accurate counting, was assessed qualitatively (presence/absence) for each condition and time point.

Impact on Sporangiophore Development:

Sporulation of *P. viticola* was directly observed on leaf discs using a 3D digital microscope (Keyence VHX-7000, Keyence Corporation, Japan) at magnifications ranging from ×100 to ×400. Images were acquired with VHX-H4M software version 1.3 (Keyence Corporation, Japan) and adjusted only for brightness and contrast. Observations were performed on at least six leaf discs per condition and were representative of the observed results.

Impact on Mycelial Network Development:

The same leaf discs were observed by epifluorescence microscopy using an Olympus BX43 microscope equipped with U/B/G filters (Olympus Corporation, Tokyo, Japan), with image acquisition performed using Infinity Analyze software version 7.1 (Lumenera Corporation, Ottawa, ON, Canada). Observations focused on discs treated with 100 mg·L^{-1} sakuranetin, a concentration which had a marked effect that was easily detectable by fluorescence microscopy. Infected discs were decolorized in 80% ethanol (10 min, 120 °C), rinsed with distilled water, treated with 1 M NaOH (30 min, 60 °C), and rinsed for 1 h in distilled water. Additional clarification was carried out with 4% NaClO (10–15 min, 60 °C), with intermediate water rinses. Discs were stained with aniline blue (0.1 g·100 mL^{-1} in 0.1 M KH_2PO_4) for 10 min, mounted in distilled water between a slide and coverslip, and examined under epifluorescence microscopy using a ×4 objective.

S4.3.2. Evaluation of the Elicitor Potential of Sakuranetin on Acclimatised Plantlets

Treatment and Conditions:

This experiment was conducted simultaneously and under the same conditions as described in Section 2.2.3, using the same plant material. Bion® (Syngenta), containing 500 g·kg−1 acibenzolar-S-methyl, was used as a positive control at 2 g·L−1, according to

the manufacturer's recommendations. Leaves number 3 and 4 below the apex were sampled 4 days after treatment in non-inoculated plants. For inoculated plants, *P. viticola* inoculation was performed 24 h after treatment, and leaves were sampled 3 days after inoculation, corresponding to 4 days after treatment. Only plants from the elicitor effect block were used to quantify the elicitation potential. Samples were immediately frozen in liquid nitrogen. This sampling time was selected to evaluate the persistence of the elicitor effect several days after application under conditions closer to vineyard practices [76].

Gene Expression Monitoring by RT-qPCR:

Total RNA was extracted from 50 mg of leaf powder ground in liquid nitrogen using PureLink™ Plant RNA Reagent (Invitrogen, Carlsbad, CA, USA), according to the manufacturer's instructions. RNA was reverse transcribed into cDNA using the Verso™ cDNA Synthesis Kit (Thermo Scientific), with reverse transcription efficiency verified by control PCR. Expression of five defense-related genes (VvSTS1, *VvPR1*, *VvCHS*, *VvCHI* and *VvPOX4*) was quantified by RT-qPCR, using *VvEF1a*, *VvUBE2* and VvACT7 as reference genes. Each biological replicate was analyzed in triplicate. RT-qPCR was performed on a CFX Opus 384 thermocycler (Bio-Rad) using SYBR Green chemistry, and relative expression levels were calculated using the ΔΔCq method after normalization to reference genes.

S4.4. Statistics

Statistical analyses were performed using GraphPad Prism 10 for macOS (v10.6.0). Data were assessed for normality, and non-parametric tests were applied when assumptions were not met. *P. viticola in vitro* data were analyzed using the Kruskal–Wallis test, whereas *B. cinerea* data were analyzed by ANOVA followed by Tukey's post hoc test. IC_{50} values were determined using a four-parameter logistic (4PL) regression model fitted to $\log_{10}$-transformed inhibitor concentrations, according to the following equation:

$$Y = Bottom + \frac{Top - Bottom}{1 + 10^{(log_{10}(IC_{50}) - X) \times HillSlope}}$$

where Bottom and Top represent the minimum and maximum responses, X the $\log_{10}$ inhibitor concentration, and HillSlope the curve slope. The IC_{50} was extracted from the fitted $\log_{10}(IC_{50})$, and its 95% confidence interval (CI) was estimated using the profile likelihood method. Because the model was fitted on log-transformed IC_{50} values, the standard deviation of $\log_{10}(IC_{50})$ was derived from the CI assuming an approximately normal distribution.

$$SD_{log_{10}(IC_{50})} = \frac{CI_{95\%}^{upper} - CI_{95\%}^{lower}}{2 \times 1.96}$$

Error propagation to the linear scale was then applied to obtain the SD of IC_{50}:

$$SD_{IC50} \approx IC50 \times \ln(10) \times SD_{\log IC50}$$

This approach expresses variability on the linear IC_{50} scale while remaining consistent with the non-linear regression model. Zoospore release data were analyzed using the Šidák multiple-comparison test, *P. viticola* infection intensity *in planta* using the Kruskal–Wallis test, and gene expression data by ANOVA followed by Dunnett's test versus the control. Differences were considered significant at $p < 0.05$.
